# FEZF1-AS1/miR-107/ZNF312B axis facilitates progression and Warburg effect in pancreatic ductal adenocarcinoma

**DOI:** 10.1038/s41419-017-0052-1

**Published:** 2018-01-18

**Authors:** Huilin Ye, Quanbo Zhou, Shangyou Zheng, Guolin Li, Qing Lin, Liangtao Ye, Yingxue Wang, Lusheng Wei, Xiaohui Zhao, Wenzhu Li, Zhiqiang Fu, Yimin Liu, Zhihua Li, Rufu Chen

**Affiliations:** 1 Department of Hepatopancreatobiliary Surgery, Sun Yat-sen Memorial Hospital, Sun Yat-sen University, Guangzhou, Guangdong Province China; 2 Guangdong Provincial Key Laboratory of Malignant Tumor Epigenetics and Gene Regulation, Sun Yat-sen Memorial Hospital, Sun Yat-sen University, Guangzhou, Guangdong Province China; 3 Department of Medical Oncology, Sun Yat-sen Memorial Hospital, Sun Yat-sen University, Guangzhou, Guangdong Province China; 4 Department of Radiotherapy, Sun Yat-sen Memorial Hospital, Sun Yat-sen University, Guangzhou, Guangdong Province China

## Abstract

Long non-coding RNAs (lncRNAs) play a pivotal role in pathological processes. However, little information has been published regarding the underlying functions and mechanisms of lncRNAs in pancreatic ductal adenocarcinoma (PDAC). A novel lncRNA FEZF1-AS1 and its sense-cognate gene ZNF312B were found to be highly expressed in human PDAC tissues and cell lines, which is associated with disease progression and predicts clinical outcome in PDAC patients. Of note, bioinformatics analysis, luciferase assays and RNA immunoprecipitation assays indicated that FEZF1-AS1 may act as an endogenous sponge by competing for miR-107, thereby modulating the derepression of ZNF312B. Downregulation of FEZF1-AS1 or ZNF312B significantly inhibited proliferation, colony formation, migration, and invasion of PDAC cells in vitro, whereas the miR-107 inhibitor abrogated the effect of dow-regulation of FEZF1-AS1 or ZNF312B in reducing oncogenic capacities of PDAC cells. In addition, FEZF1-AS1/miR-107/ZNF312B axis-induced promotion of PDAC cells proliferation appeared to be mediated by modulation of the apoptosis and the G1-S checkpoint. Furthermore, downregulation of FEZF1-AS1 repressed tumor growth in mouse xenograft models. In particular, our results highlight the contribution of FEZF1-AS1/miR-107/ZNF312B axis to Warburg effect maintenance of PDAC cells. Collectively, our findings demonstrate that the FEZF1-AS1/miR-107/ZNF312B axis regulatory network might provide a potential new therapeutic strategy for PDAC.

## Introduction

Pancreatic cancer (PC) is closely associated with a dismal prognosis, highlighted by the close parallel between disease incidence and mortality^1^. PC is the fourth leading cause of cancer deaths and its overall 5-year survival rate remains as low as 6% despite 50 years of research and therapeutic development^2^. Pancreatic ductal adenocarcinoma (PDAC), which accounts for more than 80% of PC cases, remains one of the most highly lethal malignancies^3,4^. Dysregulated lncRNA expression is correlated to the development, progression, and metastasis of various cancers, such as gastric cancer, breast cancer, and PC^5–7^. In our previous research (GEO, http://www.ncbi.nlm.nih.gov/geo/, ID: GSE61166), we utilized Arraystar Human LncRNA Microarrays to explore the lncRNA expression profile in PDAC^8^. We discovered numerous differentially expressed lncRNAs between PDAC and non-tumorous tissues, among which a novel lncRNA FEZ family zinc finger 1 antisense RNA 1 (FEZF1-AS1) was remarkably increased in PDAC tissues. However, to date, there is very limited information regarding to the specific role of FEZF1-AS1 and its sense-cognate gene zinc finger protein 312B (ZNF312B) in PDAC.

Cancer cells exhibit a unique metabolic phenotype referred to as the Warburg effect, which is characterized by enhanced glycolysis and reduced oxidative phosphorylation, even in the presence of oxygen^9,10^. During the past several decades, the Warburg effect has been consistently observed in a wide spectrum of human cancers, although the underlying molecular and biochemical mechanisms are extremely complex and remain to be elucidated^11^.

In this study, we report that high FEZF1-AS1 and ZNF312B expression is a characteristic molecular change in PDAC and investigate the biological roles of FEZF1-AS1 and ZNF312B on the phenotypes of PC cells in vitro and in vivo. Moreover, a mechanistic analysis reveals that FEZF1-AS1 may function as a competing endogenous RNA (ceRNA) to regulate the expression of ZNF312B via sponging miR-107, thereby playing an oncogenic role in promoting progression and the Warburg effect in PDAC. Our present work provides the valid evidence for a positive FEZF1-AS1/ZNF312B correlation and the crosstalk among FEZF1-AS1, miR-107 and ZNF312B, shedding new light on the utilization of FEZF1-AS1/miR-107/ZNF312B axis as a potential novel therapeutic target for the treatment of PDAC.

## Results

### FEZF1-AS1 and ZNF312B are aberrantly over-expressed in PDAC tissues and cell lines

**Fig. 1:**
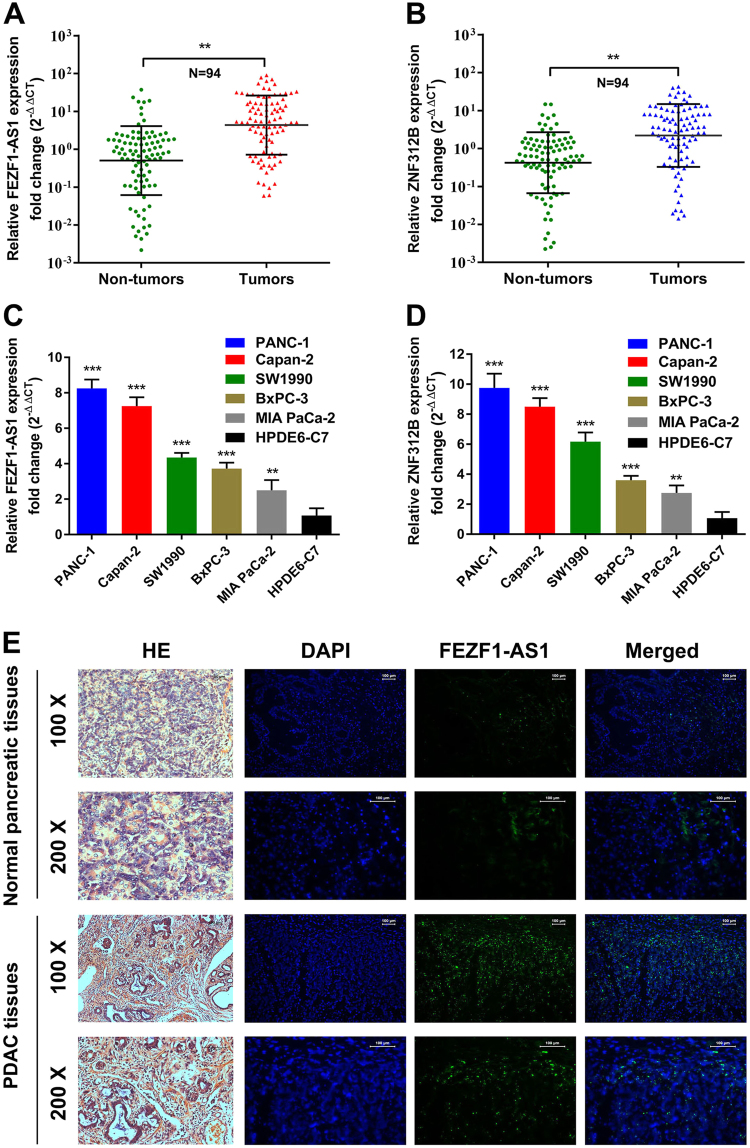
FEZF1-AS1 and ZNF312B are aberrantly over-expressed in PDAC tissues and cell lines **a**,** b** Expression levels of FEZF-AS1 and ZNF312B in 94 paired PDAC tissues and corresponding adjacent non-tumorous tissues by qRT-PCR. FEZF1-AS1 and ZNF312B expression from all tissues were normalized to GAPDH expression (∆CT) and then compared with a non-tumorous tissue and converted to the fold change (2^−∆∆CT^). **c**, **d** FEZF1-AS1 and ZNF312B expression levels were evaluated in five pancreatic cancer cell lines compared with HPDE6-C7 by qRT-PCR. Data are shown as the fold change (2^-∆∆CT^) and the mean ± SD from three independent experiments. **e** Representative images (×100 and ×200) of H&E and FISH staining for FEZF1-AS1 in paraffin-embedded PDAC and corresponding non-tumorous tissues. Paraffin-embedded tissue sections were stained using a specific probe for FEZF1-AS1 with green fluorescence. DAPI was used for nuclear counterstaining. *P < 0.05, **P < 0.01, ***P < 0.001

To identify dysregulated lncRNAs in PDAC, we previously conducted gene expression array analysis targeting 7419 lncRNAs for clinical samples from four cases of PDAC tissues and four cases of chronic pancreatitis (GEO, ID: GSE61166). From that study, we discovered that FEZF1-AS1 is one of the most upregulated lncRNAs in PDAC compared with non-tumorous tissues, suggesting a potentially crucial role for FEZF1-AS1 in PDAC tumorigenesis and development (Supplementary Fig. S1A, B). FEZF1-AS1 is a conserved 2653 bp RNA transcribed from the plus strand of chromosome 7, on the opposite strand of its cognate gene coding ZNF312B protein (7q31.32)^12^. To investigate FEZF1-AS1 and ZNF312B expression levels in PDAC, we performed quantitative real-time PCR (qRT-PCR) analysis using the total RNA extracted from 94 PDAC tissues and their matched non-neoplastic counterparts. Our current results showed that both FEZF1-AS1 and ZNF312B were significantly over-expressed in PDAC samples in comparison with those in corresponding normal tissues (Fig. 1a, b). Moreover, five PDAC cell lines (PANC-1, Capan-2, SW1990, BxPC-3, and MIAPaCa-2) showed significantly higher FEZF1-AS1 and ZNF312B levels than the pancreatic ductal epithelium cell line HPDE6-C7, with the highest expression levels observed in PANC-1 and Capan-2 cells (Fig. 1c, d). To further confirm the over-expression of FEZF1-AS1 in PDAC tissues, fluorescent in situ hybridization (FISH) was used. The data showed that specific FEZF1-AS1 positive staining primarily scattered in the cytoplasm of PDAC cells in 52 of 60 cases, whereas positive staining was occasionally observed in normal pancreatic ductal epithelia in 8 of 60 cases (*n* = 60, Fig. 1e). These results manifest that FEZF1-AS1 and ZNF312B are increased in both PDAC tissues and cell lines.

### FEZF1-AS1 over-expression is associated with clinicopathological characteristics and prognosis of PDAC patients

To examine whether FEZF1-AS1 expression levels determine the clinical progression and outcome of PDAC patients, we next assessed the clinical significance of FEZF1-AS1 in PDAC using expression levels obtained from qRT-PCR data of a cohort of 94 patients. The results indicated that dramatically high levels of FEZF1-AS1 were present in patients with poor differentiation, advanced AJCC stage, and neural invasion (Table 1). Howbeit, there was no statistical correlation between FEZF1-AS1 expression and other clinicopathological characteristics. What’s more, survival analysis revealed that FEZF1-AS1 over-expression were significantly correlated with shorter overall survival ((OS) (log-rank test, *P* < 0.001, Supplementary Fig. S2A)) with median survival times of 10 and 18 months, respectively.

**Table 1:** Correlation of FEZF1-AS1 expression and clinicopathological characteristics in patients with pancreatic ductal adenocarcinoma

Parameter		FEZF1-AS1 expression		*P*-value
		Lower (*n* = 47)	Higher (*n* = 47)	
Age (year)	<60	21	19	0.677
	≥60	26	28	—
Gender	Male	24	27	0.535
	Female	23	20	—
Differentiation	Well	25	8	—
	Moderate	15	22	—
	Poor	7	17	<0.001^a,b^
AJCC stage	I	28	8	—
	II	8	16	—
	III	10	18	—
	IV	1	5	<0.001^a,b^
Tumor range	Confined	32	26	—
	Invasion of adjacent organ/vessels/lymph node	13	17	—
	Distal metastasis	2	4	0.185^b^
Neural invasion	Negative	26	5	—
	Positive	21	42	<0.001^**a**^

*AJCC* American Joint Committee on Cancer

^a^Denotes statistical significance

^b^Kruskal–wallis one-way ANOVA test. All the other *P*-values were calculated by Pearson Chi-square test

### ZNF312B over-expression correlates with clinicopathological characteristics and prognosis of PDAC patients

To investigate the clinical relevance of the FEZF1-AS1/ZNF312B axis in PDAC, we measured the expression levels of ZNF312B protein in 94 paraffin-embedded human PDAC samples by immunohistochemistry (IHC). As described in the Methods, the expression of ZNF312B was evaluated in terms of intensity and percentage separately, and finally expressed as a score of 0, 1, 2 or 3. Representative images of score 0–3 are shown in Supplementary Fig. S2C. Statistical analysis confirmed that ZNF312B over-expression was correlated with advanced AJCC stage and neural invasion (Table 2). Furthermore, survival analysis displayed that higher ZNF312B staining intensity correlated with a poorer prognosis in PDAC patients (log-rank test, *P* < 0.001, Supplementary Fig. S2B). Moreover, the univariate analysis revealed that large tumor range and size, poor differentiation, advanced AJCC stage, neural invasion, high FEZF1-AS1 and ZNF312B expression were significantly associated with an increased risk of cancer-related death. The multivariate analysis demonstrated that advanced AJCC stage, neural invasion, high FEZF1-AS1 and ZNF312B expression levels were pivotal prognostic factors (Table 3). Of note, high expression levels of FEZF1-AS1 and ZNF312B were significantly associated with poorer survival in PDAC patients independently of the advanced AJCC stage and perineural invasion (*P* < 0.001, *P* < 0.001, Table 1; *P* < 0.001, *P* = 0.016, Table 2).

**Table 2:** Correlation between ZNF312B expression and clinicopathological characters in patients with pancreatic ductal adenocarcinoma

Parameter		Total	ZNF312B IHC scores				*P-*value
		*n* = 94	0 (*n* = 15) 1 (*n* = 22) 2 (*n* = 31) 3 (*n* = 26)				
Age (year)	Mean (range)	55.3 (37–75)	54.1 (3–0)	56.3 (4–2)	55.9 (3–5)	57.2 (5–3)	0.171^a^
Gender	Male	51	8	12	14	17	—
	Female	43	7	10	17	9	0.493
Tumor range	Confined	58	9	14	25	10	—
	Invasion of adjacent organ/vessles/lymph node	30	6	7	5	12	—
	Distal metastasis	6	0	1	1	4	0.112
Differentiation	Well	33	4	14	7	8	—
	Moderate	37	5	6	17	9	—
	Poor	24	6	2	7	9	0.505
AJCC stage	I	36	9	17	7	3	—
	II	24	3	4	10	7	—
	III	28	3	1	13	11	—
	IV	6	0	0	1	5	<0.001^b^
Neural invasion	Negative	31	11	4	11	5	—
	Positive	63	4	18	20	21	0.016^b^

*AJCC* American Joint Committee on Cancer

^a^Spearman rank correlation test. All the other *P*-values were calculated by Kruskal–Wallis one-way ANOVA test

^**b**^Denotes statistical significance

**Table 3:** Univariate and multivariate analysis of prognostic factors influencing overall survival in 94 patients with pancreatic ductal adenocarcinoma undergoing surgery

Parameter	Univariate analysis			Multivariate analysis		
	HR	95% CI	*P*-value	HR	95% CI	*P*-value
Age (years, continuous)	1.2	0.7–2.6	0.51	—	—	—
Gender						
Male (ref)	—	—	—	—	—	—
Female	0.81	0.6–1.3	0.183			
Tumor location						
Uncinate/Head (ref)	—	—	—	—	—	—
Body/Tail	0.475	0.138–1.016	0.054	—	—	—
Tumor range						
Localized (ref)	—	—	—	—	—	—
Regionally advanced	3.3	1.79–7.36	—	2.6	0.9–5.77	0.74
Metastatic	4.2	2.74–11.63	0.025^a^	3.9	0.79–10.3	0.13
Tumor size (cm, continuous)	2.2	1.9–5.1	<0.01^a^	1.96	0.92–4.3	0.51
Differentiation						
Well (ref)						
Moderate	2.1	1.3–4.1	—	1.4	0.58–3.92	0.21
Poor	8.7	4.5–17.3	<0.001^a^	6.6	0.96–14.1	0.061
AJCC stage						
I (ref)	—	—	—	—	—	—
II	1.9	0.5–11.4	—	1.73	0.51–9.9	0.31
III	4.0	1.2–18.4	—	4.1	1.3–16.2	0.019^a^
IV	9.7	3.9–66.1	<0.001^a^	9.4	3.8–54.0	<0.001^a^
Neural invasion						
Negative (ref)						
Positive	5.77	1. 498–16.51	0.0014^a^	5.82	1.56–15.5	<0.001^a^
ZNF312B expression						
Score 0						
Score 1	1.58	0.66–4.22	—	1.57	0.61–4.14	<0.01^a^
Score 2	3.0	0.4–21.1	—	3.5	0.77–22.0	0.0015^a^
Score 3	8.45	5.27–34.9	<0.001^a^	8.33	5.1–32.8	<0.001^a^
FEZF1-AS1 expression (continuous)	7.04	4.4–26.3	<0.001^a^	7.7	4.55 –26.0	<0.001^a^

*HR* hazard ratio, *CI* confidence interval, *ref* reference, *AJCC* American Joint Committee on Cancer

^a^Denotes statistical significance

### Prognostic nomogram with calibration and validation analysis

A nomogram that incorporated the AJCC classification with significant prognostic factors—neural invasion, ZNF312B expression, and FEZF1-AS1 expression was established (Supplementary Fig. S3A). The nomogram illustrated that AJCC stage and ZNF312B expression shared the largest contribution to prognosis, followed by FEZF1-AS1 expression. Neural invasion showed a moderate impact on survival. The calibration plots presented preferable agreements in the prognostic model between the nomogram prediction and actual observation for 3-year and 5-year OS (Supplementary Fig. S3B, C).

### FEZF1-AS1 primarily localizes in the cytoplasm and may correlate with ZNF312B expression

**Fig. 2:**
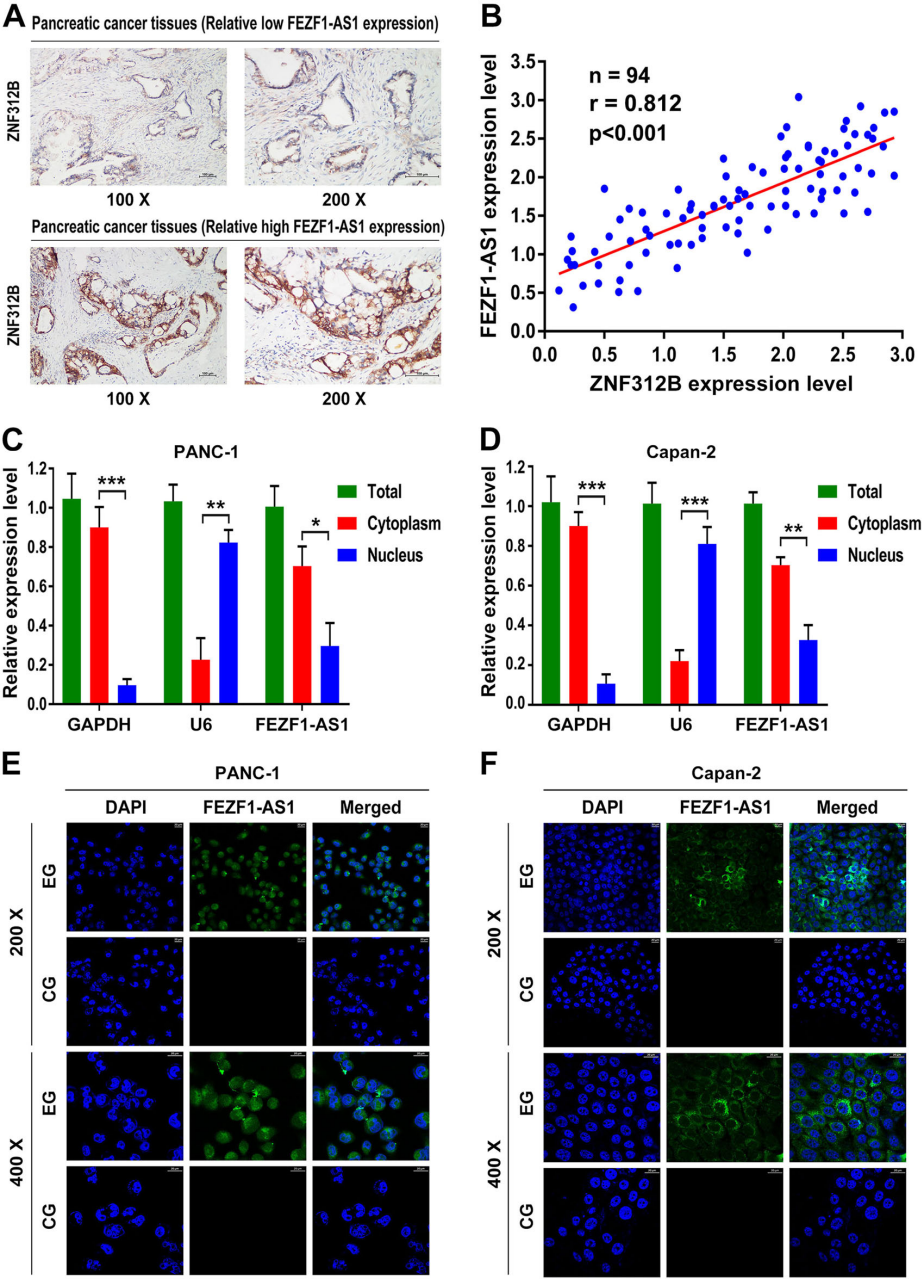
FEZF1-AS1 primarily localizes in the cytoplasm and may correlate with ZNF312B expression **a** Representative images of ZNF312B staining in PDAC tissues with relatively low FEZF1-AS1 expression and high FEZF1-AS1 expression. **b** The expression of ZNF312B positively correlated with that of FEZF1-AS1 in PDAC tissues (*n* = 94, *r* = 0.812, *P* < 0.01). **c**,** d** Real-time PCR analysis of fractionated nuclear and cytoplasmic RNA revealed that FEZF1-AS1 primarily localized in the cytoplasm. GAPDH RNA served as a positive control for cytoplasmic gene expression and U6 RNA served as a positive control for nuclear gene expression. **e**,** f** FISH assay showed specific staining for FEZF1-AS1 in the cytoplasm of PANC-1 and Capan-2 pancreatic cancer cells. *P < 0.05, **P < 0.01, ***P < 0.001

To gain further insights into the relationship between FEZF1-AS1 and ZNF312B, we examined expression levels of FEZF1-AS1 and ZNF312B in previous 94 PDAC tissues by IHC and qRT-PCR analysis. The IHC staining results revealed strong expression of ZNF312B protein in 61% (*n* = 57) of the 94 PDAC samples, and of which 74% displayed high FEZF1-AS1 expression (Fig. 2a). Bivariate correlation analysis showed that expression of ZNF312B was positively correlated with FEZF1-AS1 transcript level in PDAC tissues (*r* = 0.812, *P* < 0.001, Fig. 2b). Real-time PCR analysis of fractionated nuclear and cytoplasmic RNA revealed that lncRNA FEZF1-AS1 was primarily localized in the cytoplasm, providing a prerequisite for the interaction between FEZF1-AS1 and ZNF312B (Fig. 2c, d). In addition, FISH assay was performed on PANC-1 and Capan-2 cells and the results showed that FEZF1-AS1 was mainly located in the cytoplasm, which is in accordance with the FISH results of PDAC tissues (Fig. 2e, f).

### FEZF1-AS1 acts as a competing endogenous RNA by directly binding to miR-107

**Fig. 3:**
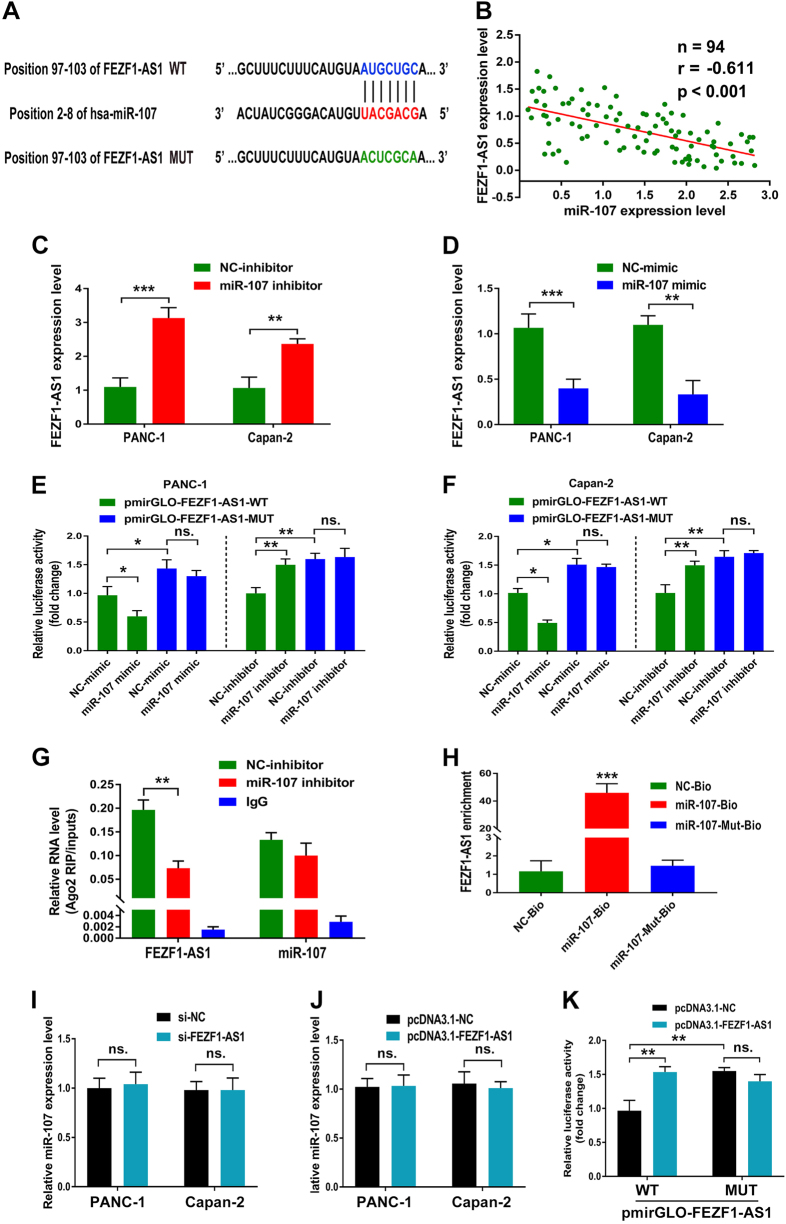
FEZF1-AS1 acts as a competing endogenous RNA by directly binding to miR-107 **a** Bioinformatics analysis showed that miR-107 might target FEZF1-AS1 and the potential wild-type-binding site and mutation site are shown. **b** Spearman correlation analysis suggested a negative relationship between FEZF1-AS1 and miR-107 in PDAC specimens (*n* = 94, *r* = −0.611, *P* < 0.01). **c**,** d** Relative FEZF1-AS1 levels were investigated in PANC-1 and Capan-2 cells after transfected with miR-107 inhibitor or miR-107 mimic. **e**,** f** Dual-luciferase assay showed a significant decrease in reporter activity after co-transfection of pmirGLO-FEZF1-AS1-WT and miR-107 mimic compared with transfection of NC-mimic, while there was no difference in reporter activity after the binding site was mutated. Conversely, inhibition of miR-107 induced a remarkable increase in luciferase activity in both cell types, which was also abrogated by binding site mutation. **g** Associations of FEZF1-AS1, miR-107 and Ago2. The amount of FEZF1-AS1 and miR-107 enriched by Ago2 or IgG was measured by qRT-PCR in the presence of miR-107 inhibitor or NC inhibitor. **h** PANC-1 cells were transfected with biotinylated WT miR-107 (miR-107-Bio) or biotinylated mutant miR-107 (miR-107-Mut-Bio) or biotinylated NC (NC-Bio). Cells were collected for biotin-based pulldown assay after 48 h of transfection. FEZF1-AS1 expression levels were analyzed by qRT-PCR. **i–j** The relative miR-107 levels in PANC-1 and Capan-2 after transfection of si-FEZF1-AS1 or pcDNA3.1-FEZF1-AS1 was investigated by qRT-PCR. **k** Luciferase assays of 293T cells transfected with pmirGLO-FEZF1-AS1-WT or pmirGLO-FEZF1-AS1-MUT reporter and pcDNA3.1-FEZF1-AS1 vector. *P < 0.05, **P < 0.01, ***P < 0.001

**Fig. 4:**
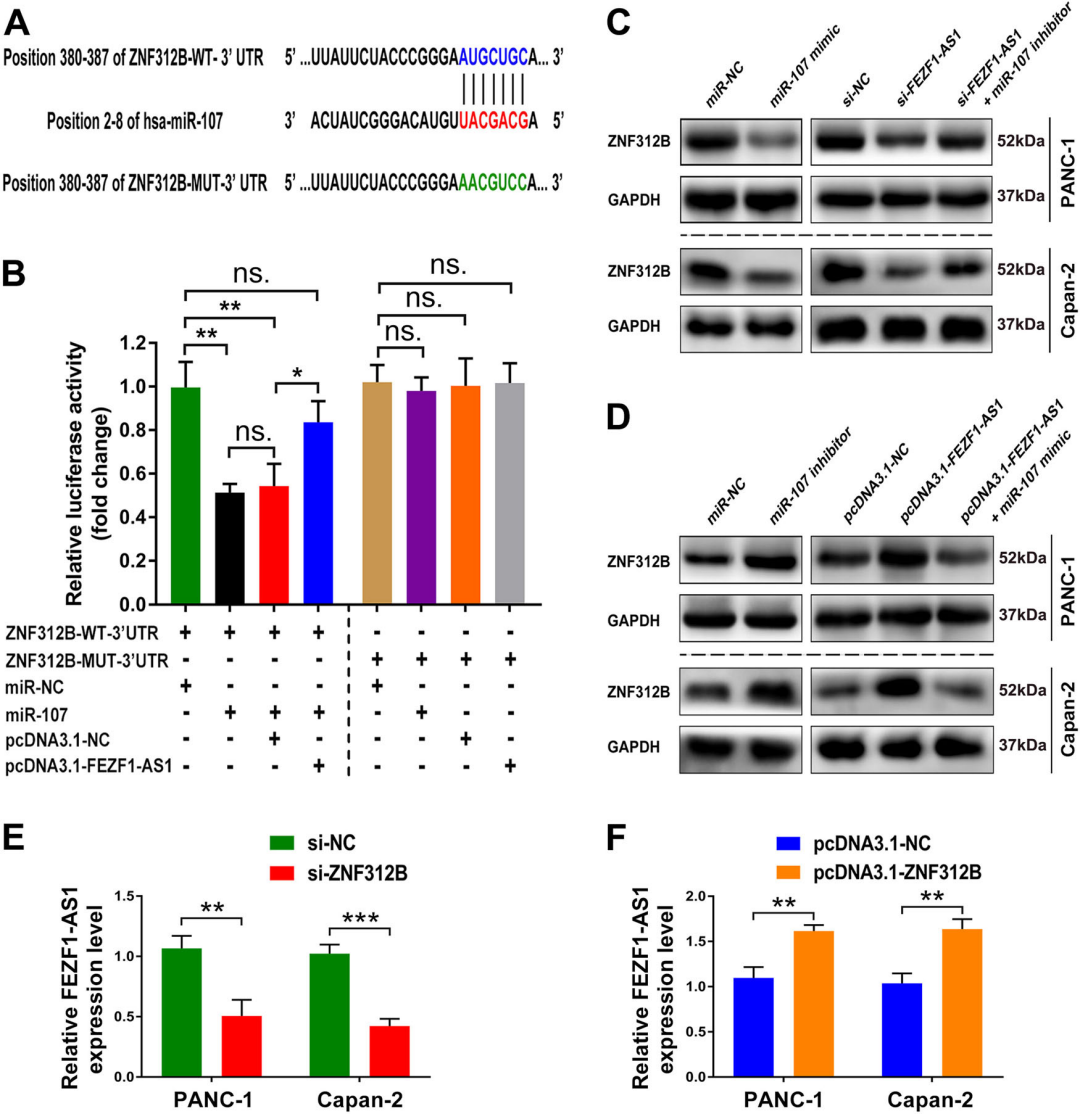
ZNF312B is a target gene of miR-107 and is regulated by FEZF1-AS1 **a** Bioinformatics analysis showed that miR-107 might target ZNF312B and the potential wild-type-binding site and mutation site are shown. **b** The 3'-UTR of ZNF312B with wild-type (ZNF312B-WT-3'UTR) or mutant site (ZNF312B-MUT-3’UTR) fused to the luciferase coding region and transfected in 293T cells with miR-107 mimic or negative control to confirm ZNF312B is the target of miR-107. ZNF312B-WT-3'UTR or ZNF312B-MUT-3'UTR and miR-107 mimic were co-transfected into 293T cells with plasmids expressing FEZF1-AS1 or with a control vector to verify the ceRNA activity of FEZF1-AS1. Histogram indicates the values of luciferase measured 48 h after transfection. **c**, **d** The effect of FEZF1-AS1 expression on endogenous ZNF312B protein in combination with the modulation of miRNA or lncRNA levels was monitored using different approaches. Cellular protein was isolated from the transfected cells 48 h later and used for western blotting. **e** Relative FEZF1-AS1 expression level in PANC-1 and Capan-2 after transfection of si-NC or si-ZNF312B. **f** Relative FEZF1-AS1 expression level in PANC-1 and Capan-2 after transfection of pcDNA3.1-NC and pcDNA3.1-ZNF312B. *P < 0.05, **P < 0.01, ***P < 0.001

Accumulating evidence has shown that lncRNAs contain motifs with sequence complementary to miRNAs and that miRNAs have an inhibition effect on lncRNAs expression and activity^13^. Considering that FEZF1-AS1 is predominantly localized in the cytoplasm of PDAC cells and inspired by the “competitive endogenous RNAs” regulatory network, we hypothesized that FEZF1-AS1 may serve as a ceRNA to regulate ZNF312B expression. Using DIANA-LncBase, Annolnc, TargetScan and miRcode bioinformatics algorithms, we found a potential miRNA candidate miR-107 targeting both FEZF1-AS1 and ZNF312B. Thus, we chose miR-107 as a model miRNA for further studies. The predicted sites of miR-107 binding to FEZF1-AS1 or ZNF312B sequence were illustrated in Fig. 3a and Fig. 4a. Spearman correlation analysis suggested a negative relationship between FEZF1-AS1 and miR-107 in 94 PDAC specimens (*r* = −0.611, *P* < 0.001, Fig. 3b). To further assess the potential relationship between FEZF1-AS1 and miR-107, we transfected PANC-1 and Capan-2 cells with miR-107 inhibitor or mimic to decrease or increase miR-107 expression level, respectively. Inhibition of miR-107 expression resulted in a significant upregulation of FEZF1-AS1 (>twofold, Fig. 3c), while miR-107 mimic significantly reduced FEZF1-AS1 expression level (>50%, Fig. 3d) in both cell lines. These results suggested that miR-107 negatively regulates FEZF1-AS1 expression either directly or indirectly.

We next sought to examine whether miR-107-mediated FEZF1-AS1 regulation occurs through direct targeting of miRNA-binding sites in the FEZF1-AS1 sequence. We subcloned full-length FEZF1-AS1 into the pmirGLO dual-luciferase reporter vector and performed luciferase assays in PANC-1 and Capan-2 cells, both of which express endogenous miR-107. As shown in Fig. 3e, f, co-transfection of PANC-1 and Capan-2 cells with pmirGLO-FEZF1-AS1-WT vector and miR-107 mimic significantly reduced luciferase reporter activity compared with the negative control (*P * < 0.05). This repressive effect was abolished by directed mutagenesis of the miR-107-binding seed region in FEZF1-AS1. Conversely, miR-107 inhibitor induced a remarkable increase in luciferase activity in both cell types (*P * < 0.01), which was also abrogated by binding site mutation. Furthermore, cells co-transfected with pmirGLO-FEZF1-AS1-MUT vector and negative control exhibited a higher level of luciferase activity with respect to the group co-transfected with WT vector and negative control, which may be due to the disruption of miR-107 binding in the MUT construct (>50%, Fig. 3e, f). Previous studies have demonstrated that miRNAs are present in the form of miRNA ribonucleoprotein complexes that contain Ago2, the vital component of the RNA-induced silencing complex (RISC)^14^. Our RNA immunoprecipitation (RIP) assays revealed that while FEZF1-AS1 was detected in Ago2 immunoprecipitates from the control group, its levels were significantly reduced in Ago2 complexes purified from cells treated with miR-107 inhibitor (*P * < 0.01, Fig. 3g), indicating that FEZF1-AS1 is likely in the miR-107–RISC complex. We further applied a biotin-avidin pulldown system to assay whether miR-107 could pulldown FEZF1-AS1. FEZF1-AS1 was pulled down as analyzed by qRT-PCR, but the introduction of mutations that disrupt base pairing between miR-107 and FEZF1-AS1 abrogated the ability of miR-107 to pull down FEZF1-AS1 (*P * < 0.001, Fig. 3h), suggesting that miR-107 interacts with FEZF1-AS1 in a sequence-specific manner. Collectively, these results demonstrate that miR-107 exerts inhibitory effects on FEZF1-AS1 expression via directly targeting FEZF1-AS1.

To further investigate the effect of FEZF1-AS1 on miR-107, we transfected si-FEZF1-AS1 or pcDNA3.1-FEZF1-AS1 vector into PANC-1 and Capan-2 cells. Nevertheless, we observed no obvious changes in miR-107 levels following FEZF1-AS1 knockdown or over-expression (Fig. 3i, j). Luciferase assays of 293T cells co-transfected with pmirGLO-FEZF1-AS1 vector and pcDNA3.1-FEZF1-AS1 vector revealed that FEZF1-AS1 over-expression induced a dramatic increase in luciferase activity, which was abrogated by miR-107-binding site mutation, suggesting that ectopically expressed FEZF1-AS1 specifically sequestered endogenous miR-107, thereby preventing it from inhibiting luciferase expression (Fig. 3k). In conclusion, our results indicate that FEZF1-AS1 is an inhibitory target of miR-107 in PDAC progression.

### ZNF312B is a target gene of miR-107 and is regulated by FEZF1-AS1

As mentioned above, we discovered a potential miRNA candidate miR-107 targeting ZNF312B via using TargetScan and miRcode software. ZNF312B was predicted to harbor miR-107 based on miRNA seed sequence matching (Fig. 4a). To confirm this prediction, we first constructed luciferase reporter plasmids harboring either the wild-type 3'-UTR of ZNF312B or a mutant 3'-UTR predicted to be insensitive to miR-107. We transfected 293T cells with the luciferase reporter plasmids, together with miR-107 mimic. Our data showed that miR-107 mimic significantly reduced the luciferase activity of the wild-type 3'-UTR, but not the mutant 3'-UTR of ZNF312B in 293T cells (*P * < 0.01, Fig. 4b). To further explore whether ZNF312B was upregulated by FEZF1-AS1, we subsequently transfected 293T cells with the luciferase reporter plasmids together with miR-107 mimic and pcDNA3.1-FEZF1-AS1. Luciferase assays indicated that, in the presence of FEZF1-AS1, the reduced luciferase activity of the wild-type 3'-UTR of ZNF312B caused by miR-107 was partly restored compared with the pcDNA3.1-NC control group (*P * < 0.05, Fig. 4b).

Furthermore, the effect of FEZF1-AS1 expression on endogenous ZNF312B protein in combination with the modulation of miRNA was monitored via the different approaches shown in Fig. 4c, d. Western blot analysis showed that miR-107 mimic or FEZF1-AS1 knockdown triggered a significant silencing effect on endogenous ZNF312B protein expression. We co-transfected PANC-1 and Capan-2 cells with si-FEZF1-AS1 and miR-107 inhibitor, and found that inhibition of miR-107 partly abrogated the silencing effect of FEZF1-AS1 knockdown on endogenous ZNF312B protein expression (Fig. 4c). Accordingly, miR-107 inhibitor or FEZF1-AS1 over-expression dramatically elevated endogenous ZNF312B protein expression. In addition, we co-transfected PANC-1 and Capan-2 cells with pcDNA3.1-FEZF1-AS1 and miR-107 mimic, and found that miR-107 mimic partly abrogated the promoting effect of FEZF1-AS1 over-expression on endogenous ZNF312B protein expression (Fig. 4d). Moreover, compared with negative control groups, ZNF312B downregulation significantly reduced FEZF1-AS1 expression in PANC-1 and Capan-2 cells (Fig. 4e), while ZNF312B over-expression dramatically elevated FEZF1-AS1 expression (Fig. 4f). Taken together, these data indicate that FEZF1-AS1 acts as an endogenous sponge by sequestering miR-107 and thus abolishing the miRNA-induced repressing effect on the ZNF312B 3'-UTR.

### FEZF1-AS1/miR-107/ZNF312B pathway facilitates proliferation and inhibits apoptosis of PDAC cells in vitro

**Fig. 5:**
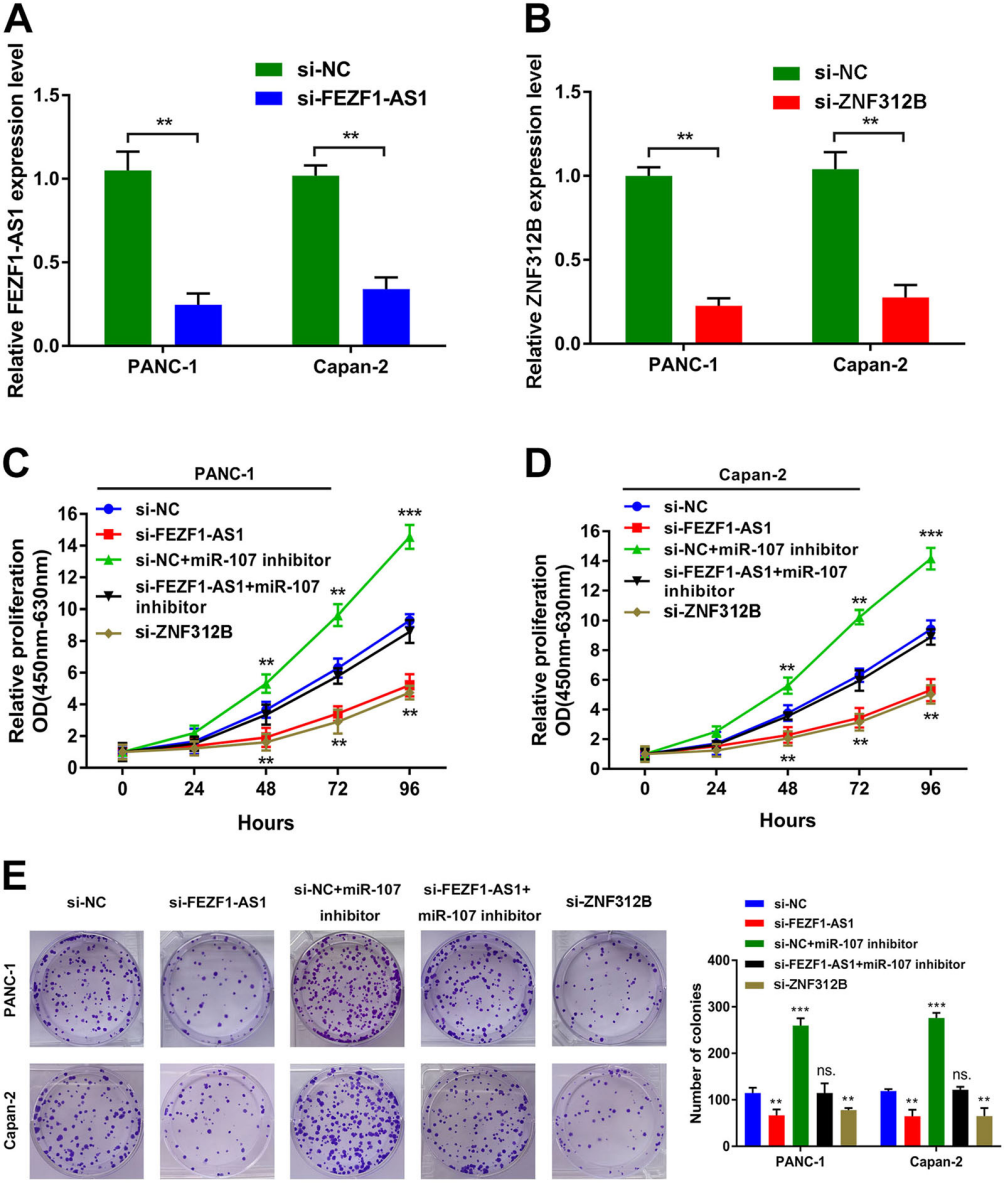
Effects of FEZF1-AS1 or ZNF312B knockdown on the inhibition of PDAC cell proliferation and colony formation could be rescued by miR-107 inhibitor in vitro **a**,** b** The expression of FEZF1-AS1 or ZNF312B was suppressed by specific siRNAs in PANC-1 and Capan-2 cells. **c**, **d** The cell viability of si-NC, si-FEZF1-AS1, si-NC + miR-107 inhibitor, si-FEZF1-AS1 + miR-107 inhibitor, or si-ZNF312B transfected PANC-1 and Capan-2 cells by CCK-8 assay. **e** The proliferation of si-NC, si-FEZF1-AS1, si-NC + miR-107 inhibitor, si-FEZF1-AS1 + miR-107 inhibitor, or si-ZNF312B transfected PANC-1 and Capan-2 cells by colony formation assay. Values represented the mean ± SD from three independent experiments. *P < 0.05, **P < 0.01, ***P < 0.001, Student’s *t*-test

To further explore the causal role of FEZF1-AS1 and ZNF312B in PDAC progression, in vitro functional characterizations were performed. First, the qRT-PCR assay revealed that the expression of FEZF1-AS1 or ZNF312B was significantly reduced by specific siRNAs and upregulated by pcDNA3.1 cDNA vectors in PANC-1 and Capan-2 cells, correspondingly (Fig. 5a, b and Supplementary Fig. S4A, B). Compared with si-NC cells, both FEZF1-AS1 and ZNF312B downregulation greatly attenuated tumor cell proliferation, as determined by a CCK-8 assay. Besides, the miR-107 inhibitor abrogated the effect of si-FEZF1-AS1 on the reduction of cell viability of both PDAC cell lines (Fig. 5c, d). On the contrary, FEZF1-AS1 or ZNF312B over-expression had the opposite effect (Supplementary Fig. S4C, D). Consistently, the colony formation assay confirmed these findings (Fig. 5e and Supplementary Fig. S4E). Furthermore, flow cytometry analysis showed that both FEZF1-AS1 and ZNF312B downregulation remarkably increased the apoptotic rate of PANC-1 and Capan-2 cells (Supplementary Fig. S5A, B). Flow cytometry analysis further demonstrated that both FEZF1-AS1 and ZNF312B downregulation resulted in a substantial accumulation of PDAC cells in G0/G1 phase, accompanied by a substantial decrease in S phase (Supplementary Fig. S5C, D). In addition, miR-107 inhibitor significantly abolished the effect of si-FEZF1-AS1 in promoting apoptosis and inducing G1 phase arrest (Supplementary Fig. S5A–D). In contrast, FEZF1-AS1 or ZNF312B over-expression had the opposite effect on apoptosis and cell cycle distribution (Supplementary Fig. S6A–D). Thus, FEZF1-AS1/miR-107/ZNF312B axis-induced promotion of PDAC cell proliferation appeared to be mediated by modulation of the apoptosis and the G1-S checkpoint. These results demonstrate the pivotal role of FEZF1-AS1/miR-107/ZNF312B axis in the proliferation and apoptosis of PDAC cells.

### FEZF1-AS1/miR-107/ZNF312B pathway promotes PDAC cell migration and invasion in vitro

**Fig. 6:**
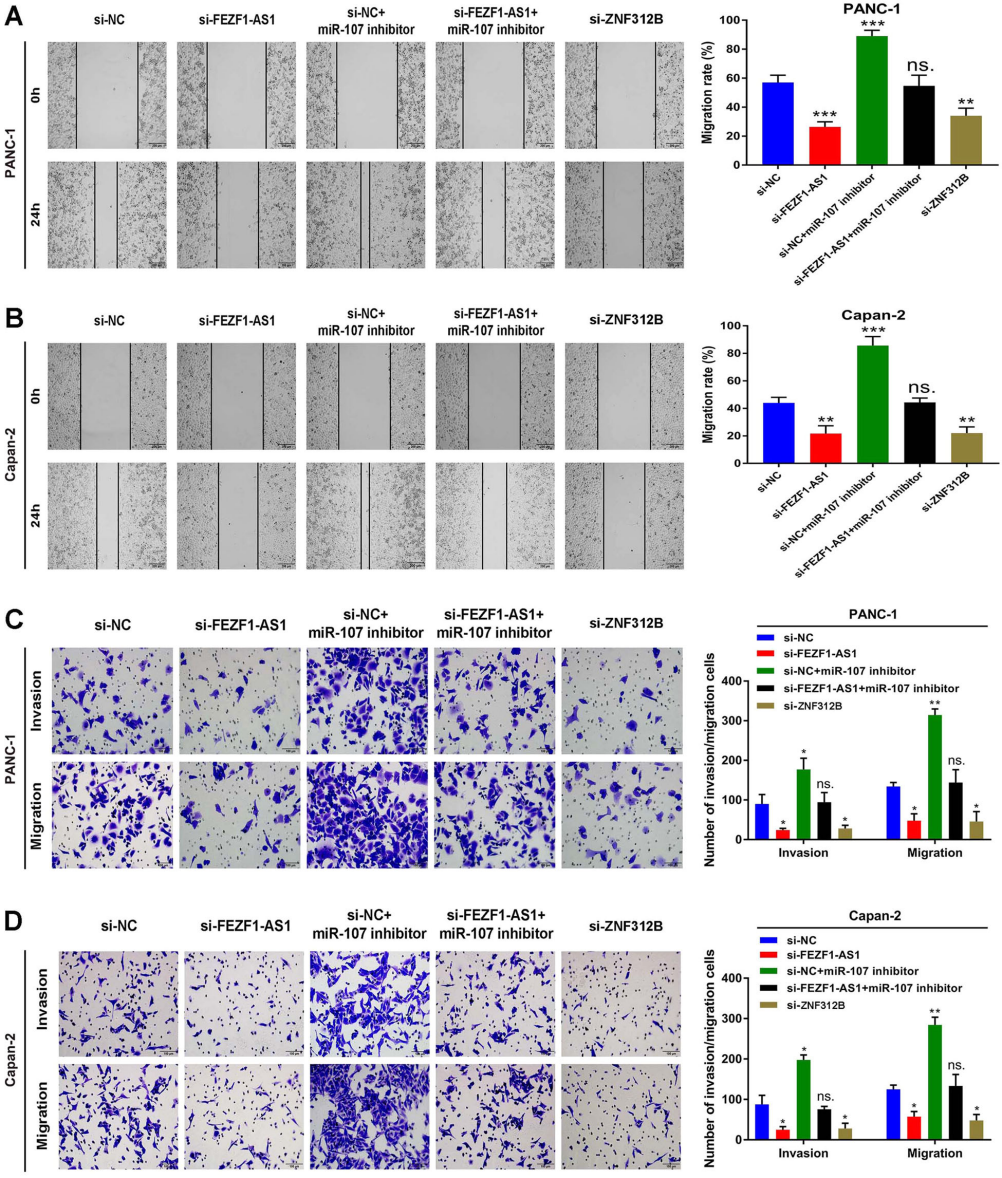
Effects of FEZF1-AS1 or ZNF312B knockdown on the inhibition of PDAC cell migration and invasion could be rescued by miR-107 inhibitor in vitro PDAC cell line PANC-1 and Capan-2 were treated as in described in Materials and Methods. **a**,** b** The motility of PANC-1 and Capan-2 cells transfected with si-FEZF1-AS1 or si-ZNF312B compared with the controls by wound healing assay. **c**,** d** The migration and invasion of PANC-1 and Capan-2 cells transfected with si-NC, si-FEZF1-AS1, si-NC + miR-107 inhibitor, si-FEZF1-AS1 + miR-107 inhibitor or si-ZNF312B compared with the controls by transwell assay. Values represented the mean ± SD from three independent experiments. *P < 0.05, **P < 0.01, ***P < 0.001, Student’s *t*-test

Enhanced cell migration and invasion abilities play a critical role in cancer progression, leading to a poor prognosis. Our wound healing scratch assay revealed that knockdown of FEZF1-AS1 or ZNF312B dramatically decreased cell motility, whereas miR-107 inhibitor abrogated the effect of si-FEZF1-AS1 on reducing cell viability (Fig. 6a, b). Conversely, over-expression of FEZF1-AS1 or ZNF312B had the opposite effect (Supplementary Fig. S7A, B). Moreover, the transwell assay also demonstrated that knockdown of FEZF1-AS1 or ZNF312B dramatically attenuated the migration and invasion of PDAC cells, while co-transfection of miR-107 inhibitor and si-FEZF1-AS1 showed that miR-107 inhibitor increased cell migration and invasion attenuated by si-FEZF1-AS1 (Fig. 6c, d). Conversely, over-expression of FEZF1-AS1 or ZNF312B significantly increased the migration and invasion of the two cell lines, while miR-107 mimic decreased cell migration and invasion promoted by pcDNA3.1-FEZF1-AS1 (Supplementary Fig. S7C, D). These observations suggest that the effects of FEZF1-AS1 or ZNF312B over-expression on the promotion of PDAC cell migration and invasion could be diminished by miR-107 mimic, in accordance with the suppression of ectopic FEZF1-AS1 and ZNF312B expression by miR-107 mimic.

### Knockdown of FEZF1-AS1 inhibits tumor growth in vivo

**Fig. 7:**
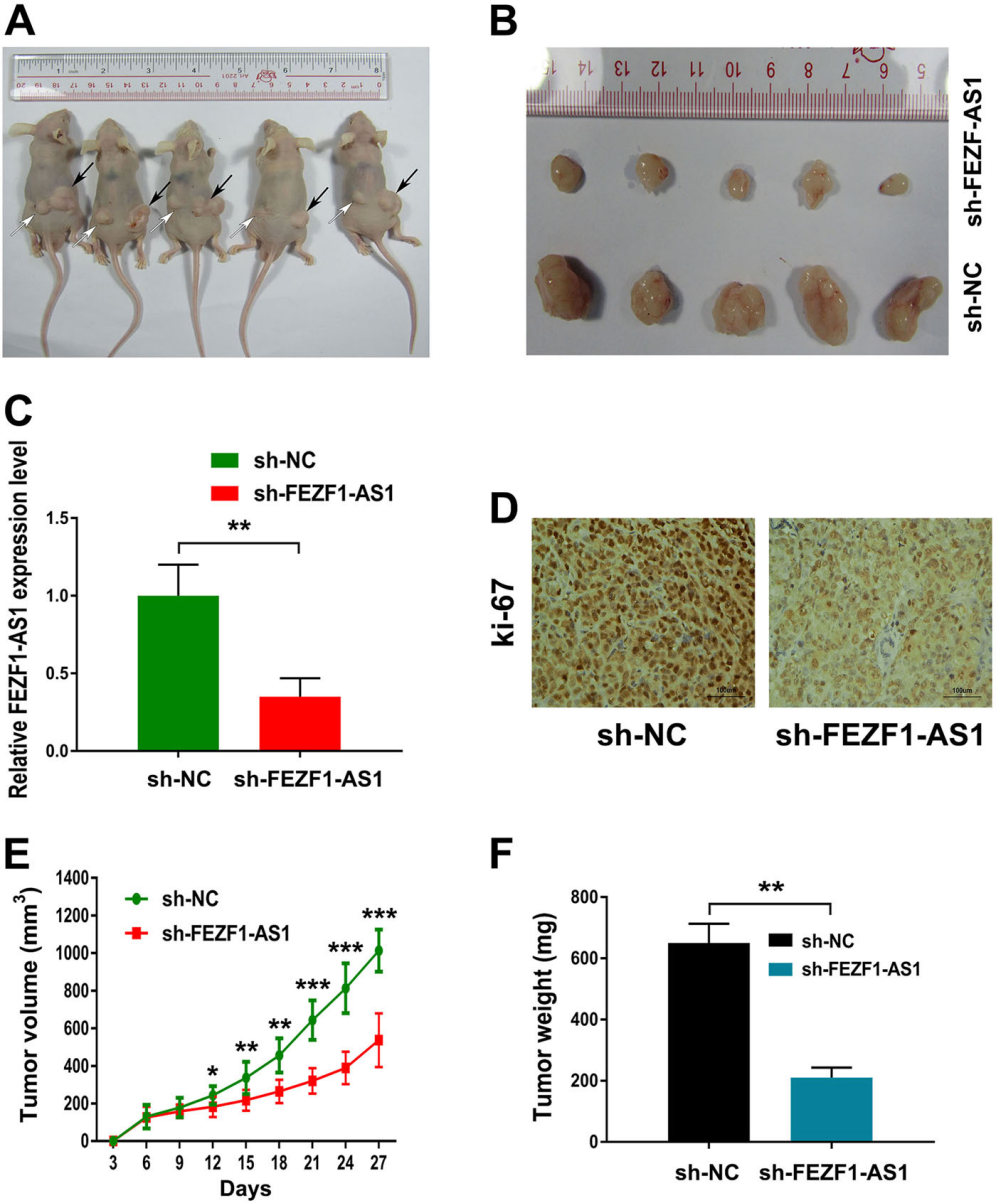
Knockdown of FEZF1-AS1 inhibits tumor growth in mouse xenograft models **a**,** b** A subcutaneous injection of PANC-1 cells with stable knockdown of FEZF1-AS1 or mock cells was administered into the subcutaneous bilateral hind leg of nude mice. At 28 days after subcutaneous injection, PANC-1 cells transfected with sh-FEZF1-AS1 (white arrow) and mock cells (black arrow) produced primary tumors and representative figure of tumor formation is shown. **c** qRT-PCR analyzed the expression of FEZF1-AS1 in tumor tissues from sh-FEZF1-AS1 PANC-1 cells compared with sh-NC PANC-1 cells. **d** Representative images (×200) of IHC staining of the tumor. The IHC staining showed that FEZF1-AS1 knockdown decreased the proliferation index Ki-67. **e** Growth curves of xenograft tumors after subcutaneous injection of mice with FEZF1-AS1-silenced PANC-1 or negative control cells. The tumor volumes were measured every 3 days after inoculation. **f** Tumor weights are shown as the means of tumor weights ±SD when the tumors were harvested. *P < 0.05, **P < 0.01, ***P < 0.001

The effect of FEZF1-AS1 on tumor growth was assessed in vivo by administering a subcutaneous injection of PANC-1 cells with stable knockdown of FEZF1-AS1 (sh-FEZF1-AS1) or mock cells (sh-NC) into the subcutaneous bilateral hind leg of nude mice. All mice developed xenograft tumors at the injection site (Fig. 7a, b). Positive staining for the proliferation marker Ki-67 was significantly decreased in FEZF1-AS1-silenced PANC-1 cells compared with control cells (Fig. 7c, d). In addition, xenograft tumors grown from FEZF1-AS1-silenced PANC-1 cells had smaller mean volumes and formed more slowly than xenograft tumors grown from control cells (Fig. 7e, f). Taken together, these findings validate that FEZF1-AS1 is an important player in the regulation of PDAC proliferation capacity in mouse xenograft models.

### FEZF1-AS1/miR-107/ZNF312B pathway maintains Warburg effect of PDAC cells

The Warburg effect, summarized as aerobic glycolysis, was a renaissance in cancer research, including PC^15^. Previous studies have shown that ZNF312B plays a key role in tumor progression and metastasis in gastric cancer via transcriptional activation of the K-ras oncogene^16^. A recent progress in the contribution of K-ras to PC oncogenesis and progression is that K-ras induces glucose metabolic reprogramming^17^. Therefore, we sought to investigate whether FEZF1-AS1/miR-107/ZNF312B axis is linked to the Warburg effect of PDAC cells.

**Fig. 8:**
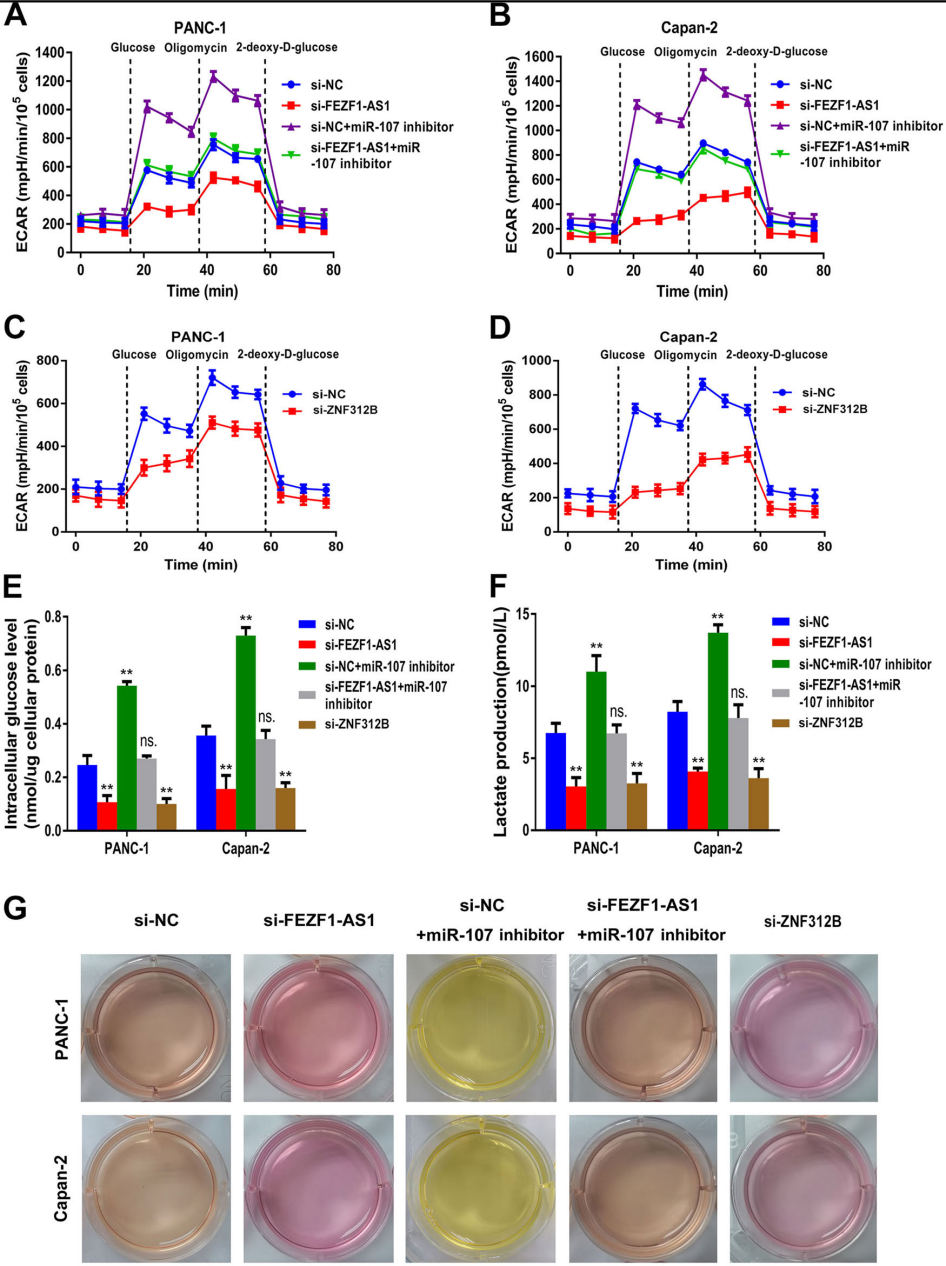
Effects of FEZF1-AS1 or ZNF312B knockdown on the inhibition of PDAC cell glycolytic capacity could be rescued by miR-107 inhibitor in vitro **a**,** b** Silencing of FEZF1-AS1 expression abrogated the glycolytic capacity of PANC-1 and Capan-2 cells, while the miR-107 inhibitor could rescue the inhibitory effects of si-FEZF1-AS1 on the glycolytic process in both PDAC cell lines, as reflected by ECAR analysis. **c**, **d** Silencing of ZNF312B expression inhibited the glycolytic capacity in PANC-1 and Capan-2 cells, as reflected by ECAR analysis. **e**, **f** Silencing of FEZF1-AS1 or ZNF312B expression inhibited the glucose uptake and lactate production in PANC-1 and Capan-2 cells, while the miR-107 inhibitor could rescued the effect of si-FEZF1-AS1 or si-ZNF312B on glucose uptake and lactate production of both PDAC cell lines. Intracellular glucose levels were measured and normalized based on protein concentration. **g** PANC-1 and Capan-2 cells-expressing si-FEZF1-AS1 or si-ZNF312B combined with miR-107 inhibitor were cultured under normoxic conditions for 24 h. Acidification of the culture medium was evaluated by visually inspecting the colour of the medium. *P < 0.05, **P < 0.01

Our data revealed that the extracellular acidification rate (ECAR) significantly decreased in FEZF1-AS1 or ZNF312B knockdown cells by applying Seahorse XF analyzers, indicating that silencing FEZF1-AS1 or ZNF312B inhibited glycolytic process in PDAC cells. In addition, miR-107 inhibitor rescued the effect of si-FEZF1-AS1 on inhibiting the glycolytic process of both PDAC cell lines (Fig. 8a, d). In contrast, FEZF1-AS1 or ZNF312B over-expression combined with miR-107 mimic had the opposite effect (Supplementary Fig. S8A–D). Accordingly, silencing FEZF1-AS1 or ZNF312B also led to a strong decrease in glucose uptake and lactate production, while FEZF1-AS1 or ZNF312B over-expression had the opposite effect. Furthermore, the effect of FEZF1-AS1 knockdown on the inhibition of glucose uptake and lactate production in PDAC cells could be rescued by miR-107 inhibitor, whereas the effect of FEZF1-AS1 over-expression on the promotion of glucose uptake and lactate production in PDAC cells could be diminished by miR-107 mimic (Fig. 8e, f and Supplementary Fig. S8E, F). Consistent with these findings, acidification of the culture medium via visually inspecting its color further validated these findings (Fig. 8g and Supplementary Fig. S8G). In conclusion, these results reinforced the contribution of FEZF1-AS1/miR-107/ZNF312B molecular axis to Warburg effect maintenance in PDAC cells.

## Discussion

Over the past decades, accumulating evidence has emphasized that the association of lncRNAs with tumor invasiveness and metastatic potential, and they have been considered diagnostic and prognostic markers for several types of cancer including PC^18–20^. However, thus far, little is known about the clinical and biological function of lncRNA FEZF1-AS1 and its sense-cognate gene ZNF312B in PDAC^12,21–25^. As reported in our work, we examined the aberrant expression of FEZF1-AS1, its association with clinicopathological characteristics and patients’ prognosis, its regulatory mechanisms on ZNF312B, and its biological effects on PC in vitro and in vivo.

It has been reported that lncRNAs can be implicated in translation, degradation, pre-messenger RNA splicing, and protein activities even as microRNA sponges in a sequence-dependent manner^26,27^. Inspired by the “competitive endogenous RNAs” regulatory network and mounting evidence indicating that lncRNAs may participate in this regulatory circuitry, we hypothesized that FEZF1-AS1 may serve as a ceRNA to modulate ZNF312B expression and we searched for potential interactions with miRNAs. Using bioinformatics analysis, luciferase assays, biotin-avidin pulldown assay and RNA immunoprecipitation assays, we confirmed that FEZF1-AS1 can act as an endogenous sponge by competing for miR-107, thereby regulating the derepression of ZNF312B.

In contrast to normal differentiated cells, which rely primarily on mitochondrial oxidative phosphorylation to generate the energy needed for cellular processes, most cancer cells instead rely on aerobic glycolysis, a phenomenon termed “the Warburg effect”^28,29^. The contribution of the FEZF1-AS1/miR-107/ZNF312B pathway to Warburg effect maintenance in PC has never been previously reported, and thus we sought to uncover the molecular mechanism underlying the FEZF1-AS1/miR-107/ZNF312B axis in glycolysis regulation. As exhibited in our results, silencing FEZF1-AS1 or ZNF312B inhibited the glycolytic process, glucose uptake and lactate production in PC cells. In addition, miR-107 inhibitor rescued the effect of si-FEZF1-AS1 on inhibiting glucose metabolism in PDAC cells. Taken together, the FEZF1-AS1/miR-107/ZNF312B axis is responsible for Warburg effect maintenance in PDAC cells, which meets the demands for continuous energy and nutrients to support uncontrolled proliferation.

In conclusion, we demonstrated that lncRNA FEZF1-AS1 and its sense-cognate gene ZNF312B are independent prognostic factors for PDAC patients and promote the progression of PDAC both in vitro and in vivo. This study provides the first connection between FEZF1-AS1/miR-107/ZNF312B axis and PDAC progression and the Warburg effect. A deeper characterization of the function and downstream signaling pathways influenced by FEZF1-AS1/miR-107/ZNF312B pathway dysregulation may provide novel insights into the underlying mechanisms of tumorigenesis and progression of PDAC. These results will help to uncover new predictive and treatment targets for PC.

## Materials and methods

### Patients and clinical samples

A total of 94 paired PDAC and corresponding non-tumorous tissues were collected from patients who underwent surgery at Sun Yat-sen Memorial Hospital between 2008 and 2015. The protocol was approved by the Hospital’s Protection of Human Subjects Committee and written informed consent was obtained from all patients before sample collection. All the cases were diagnosed with PDAC by pathologic examination of hematoxylin-eosin (HE) stained samples. None of the patients received any preoperative chemotherapy or radiotherapy. All samples were immediately snap-frozen in liquid nitrogen and stored at −80 °C until further use. The detailed clinicopathologic characteristics of the patients are summarized in Table 1. Follow-up data were carried out completely and OS was defined as the time interval between the date of surgery and the date of death or the end of follow-up (August 2015).

### Microarray analysis

Microarray analysis was performed as described in Supplementary Methods. The microarray platform and data were submitted to the Gene Expression Omnibus public database at the National Center for Biotechnology Information (GEO, http://www.ncbi.nlm.nih.gov/geo/, ID: GSE61166).

### Cell culture

Human PC cell lines (PANC-1, Capan-2, MIAPaCa-2, SW1990, and BxPC-3) and the immortal human pancreatic duct epithelial cell line (HPDE6-C7) were purchased from the American Type Culture Collection (ATCC, Manassas, USA). The procedures are briefly described in Supplementary Methods.

### RNA extraction and quantitative real-time PCR (qRT-PCR)

Total RNA was extracted from frozen PC tissues and corresponding non-neoplastic tissues or cultured cell lines using TRIzol reagent (Invitrogen, Carlsbad, USA) according to the manufacturer’s instructions. The next procedure was performed as described in Supplementary Methods. All primer sequences of qRT-PCR are listed in Table S1 (see Additional file 1). The qRT-PCR results were analyzed and shown as the fold change (2^-ΔΔCt^). For expression in tissues, the levels were first normalized to GAPDH expression as ΔCt and then compared with one of the tissues and converted to the fold change (2^-ΔΔCt^). To analyze the clinical significance of FEZF1-AS1, tissues from 94 patients were divided into two groups—the high and low expression group—depending on the fold change (2^-ΔΔCt^) of FEZF1-AS1 by using the median expression value. For analysis of the expression in cells, the levels were compared with the controls and converted to the fold change (2^-ΔΔCt^).

### Fluorescent in situ hybridization (FISH)

A locked nucleic acid (LNA) probe with complementarity to a section of FEZF1-AS1 was labeled with 5-carboxyfluorescein (FAM), which was synthesized by Biosense Bioscience Co.Ltd (Guangzhou, China). After dewaxing and rehydration, the samples were digested with proteinase K, denatured with formamide, and hybridized with the FEZF1-AS1 probe at 42 °C overnight. DAPI (4',6-diamidino-2-phenylindole) was subsequently used to counterstain the nuclei. The samples were analyzed in a drop of fluorescence decay mounting medium under a fluorescence microscope. The excitation wavelengths used were 360 nm for DAPI and 490 nm for FEZF1-AS1 with emissions detected at the appropriate wavelengths. The negative control was a scramble LNA probe. The probe sequence of FEZF1-AS1 is described in Supplementary Methods.

### Cell transfection and viral infection

For transient knockdown experiments, the small-interfering RNAs (siRNAs) were as follows: FEZF1-AS1 siRNA (si-FEZF1-AS1), ZNF312B siRNA (si-ZNF312B) and synthetic sequence-scrambled siRNA (si-NC) were purchased from GenePharma Co. (Shanghai, China). Stable suppression of FEZF1-AS1 was performed by short-hairpin RNA (sh-RNA) interference. All oligonucleotide sequences are listed in Table S2 (see Additional file 2). The details of transfection and infection procedures are described in Supplementary Methods.

### Expression construct

The sequence of FEZF1-AS1 or ZNF312B was synthesized and subcloned into pcDNA3.1 (Invitrogen, Shanghai, China). Ectopic expression of FEZF1-AS1 or ZNF312B was achieved by pcDNA3.1-FEZF1-AS1 or pcDNA3.1-ZNF312B transfection, and empty pcDNA vector (empty) was used as the control. The expression level of FEZF1-AS1 or ZNF312B was detected by qRT-PCR.

### Western blot analysis

Western blot analysis was performed as described in Supplementary Methods. Primary antibodies were rabbit anti-human ZNF312B antibody (1:1000, #ab81251, Abcam) and rabbit anti-human GAPDH antibody (1:1000, #ab18162, Abcam). GAPDH was used as a loading control. The HRP-linked secondary antibody was goat anti-rabbit IgG (1:5000; Cell Signaling Technology, Boston, USA).

### Immunohistochemistry analysis

Paraffin-embedded samples of primary carcinomas were immunostained for primary rabbit anti-human ZNF312B antibody (1:200, #ab81251, Abcam). For evaluation and grading of ZNF312B staining results, a scoring criterion previously described by Ohara et al.^30^ was used. Staining was assessed by two pathologists according to the scoring criteria. Cases with discrepancies were jointly reevaluated until a consensus was reached. These procedures are described in Supplementary Methods.

### Cell cytoplasm/nuclear fraction isolation

The cytosolic and nuclear fractions of PANC-1 or Capan-2 cells were separated using the PARIS Kit (Life Technologies) according to the manufacturer’s instructions. RNA was extracted from both fractions. Then, qRT-PCR was performed to evaluate the expression ratios of specific RNA molecules between the cytoplasmic and nuclear fractions. U6 served as the nuclear control, and GAPDH served as the cytosolic control.

### RNA-binding protein immunoprecipitation

RIP experiments were performed using the Magna RIP RNA-binding protein immunoprecipitation kit (Millipore, Billerica, MA, USA) according to the manufacturer’s instructions. Briefly, cells were collected and lysed in complete RIP lysis buffer. The cell extract was incubated with RIP buffer containing magnetic beads conjugated to a human anti-Ago2 antibody (Abcam, Cambridge, MA, USA). The samples were then incubated with proteinase K with shaking to digest proteins. Subsequently, immunoprecipitated RNA was isolated. The RNA concentration was measured using a NanoDrop spectrophotometer, and RNA quality was assessed using a bioanalyzer (Agilent). Lastly, purified RNA was subjected to qRT-PCR analysis.

### Pulldown assay with biotinylated miRNA

PANC-1 cells were transfected with biotinylated miRNA (50 nM), harvested 48 h after transfection. The cells were washed with PBS followed by brief vortexing and incubated in lysis buffer on ice for 10 min. The lysates were precleared by centrifugation and 50 ml of the samples was aliquoted for the input. The remaining lysates were incubated with M-280 streptavidin magnetic beads (Sigma). To prevent non-specific binding of RNA and protein complexes, the beads were coated with RNase-free bovine serum albumin (BSA) and yeast tRNA (Sigma). The beads were then incubated at 4 °C for 3 h and washed twice with ice-cold lysis buffer, three times with low salt buffer, and once with high salt buffer. Finally, the bound RNA were purified using TRIzol reagent (Invitrogen) for further qRT-PCR analysis.

### Dual-luciferase reporter assay

PANC-1 or Capan-2 cells were seeded at 3 × 10^4^ cells/well in 24-well plates and allowed to settle overnight. The next day, cells were co-transfected with pmirGLO-FEZF1-AS1-WT or pmirGLO-FEZF1-AS1-MUT reporter plasmids, pmirGLO-ZNF312B-WT or pmirGLO-ZNF312B-MUT reporter plasmids and miR-107 mimic or inhibitor. Twenty-four hours after transfection, the relative luciferase activity was measured using the dual-luciferase reporter assay system (Promega, Madison, WI, USA) and normalized to Renilla luciferase activity.

### Tumor formation assay

Athymic BALB/c nude mice (4–6-weeks-old) were used for the tumor formation assay. The tumor volume was calculated as follows: Volume = (*L* × *W*^2^)/2 (V: volume; L: length diameter; W: width diameter). The animal care and experimental protocols were approved by the institutional guidelines of Guangzhou Province and by the Use Committee for Animal Care. All necessary steps were taken to minimize suffering and distress of to the mice. The procedures of tumor formation assay is described in Supplementary Methods.

### Glucose uptake and lactate production assay

PDAC cells were cultured in glucose-free DMEM (Dulbecco's Modified Eagle Medium) for 16 h, and then incubated with high-glucose DMEM under normoxic conditions for an additional 24 h. The culture medium was removed, and the intracellular glucose levels were measured using a fluorescence-based glucose assay kit (BioVision) according to the manufacturer’s instructions. Lactate levels were measured using a lactate oxidase-based colorimetric assay read at 540 nm according to the manufacturer’s instructions (Beyotime, Wuxi, China) and normalized to the cell numbers.

### Extracellular acidification rate (ECAR)

The ECAR was measured using an XF^e^96 Extracellular Flux Analyzer (Seahorse Bioscience, North Billerica, MA, USA). Briefly, 5 × 10^4^ cells were seeded into XF^e^96 tissue culture plates in quadruplicate, and ECAR was measured 24 h later under basal conditions and after sequential treatment of the cells with glucose, oligomycin and 2-deoxy-d-glucose. Cells were trypsinized and counted using a Coulter counter (Beckman Coulter) and the cell count was used for normalization purposes.

### Statistical analysis

All statistical analyses were performed using SPSS Statistics 18.0 (IBM Chicago, IL, USA). The Chi-square test (*χ*^2^ test) for non-parametric variables, and Student’s *t*-test or one-way analysis of variance (ANOVA) for parametric variables were used (two-tailed). All data are presented as the mean ± SD from at least three independent experiments, unless otherwise noted. Differences in patient survival were assessed using the Kaplan–Meier method and analyzed using the log-rank test in a univariate analysis. Univariate and multivariate Cox regression analyses were performed to assess the relative risk for each factor. Correlation analysis was examined with two-sided Pearson’s correlation. Nomograms to predict the prognostic value were formulated on the basis of multivariate analysis using the rms package in R version 3.2.0 (www.r-project.org). The prediction and discrimination of the nomograms were determined using the concordance index (C-index) and assessed by comparing nomogram-predicted vs. observed Kaplan–Meier estimates of survival probability subjected to 100 bootstrap resamples. All tests were two-sided, and results with *P* < 0.05 were considered statistically significant.

Additional methods are described in Supplementary Methods.

## Electronic supplementary material

Table S1

Table S2

marked-up version of Supplementary Methods

Supplementary Figures

Figure S1

Figure S2

Figure S3

Figure S4

Figure S5

Figure S6

Figure S7

Figure S8

## References

[1] Kamisawa T, Wood LD, Itoi T, Takaori K (2016). Pancreatic cancer. Lancet.

[2] Siegel RL, Miller KD, Jemal A (2017). Cancer Statistics, 2017. CA Cancer J. Clin..

[3] Hidalgo M (2010). Pancreatic cancer. N. Engl. J. Med..

[4] Wolfgang CL (2013). Recent progress in pancreatic cancer. CA Cancer J. Clin..

[5] Wang KC, Chang HY (2011). Molecular mechanisms of long noncoding RNAs. Mol. Cell.

[6] Gibb EA, Brown CJ, Lam WL (2011). The functional role of long non-coding RNA in human carcinomas. Mol. Cancer.

[7] Tsai MC, Spitale RC, Chang HY (2011). Long intergenic noncoding RNAs: new links in cancer progression. Cancer Res..

[8] Li Z (2015). The long non-coding RNA HOTTIP promotes progression and gemcitabine resistance by regulating HOXA13 in pancreatic cancer. J. Transl. Med..

[9] WARBURG O (1956). On the origin of cancer cells. Science.

[10] DeBerardinis RJ, Thompson CB (2012). Cellular metabolism and disease: what do metabolic outliers teach us?. Cell.

[11] Zu XL, Guppy M (2004). Cancer metabolism: facts, fantasy, and fiction. Biochem. Biophys. Res. Commun..

[12] Hashimoto H (2000). Expression of the zinc finger gene fez-like in zebrafish forebrain. Mech. Dev..

[13] Liu XH (2014). Lnc RNA HOTAIR functions as a competing endogenous RNA to regulate HER2 expression by sponging miR-331-3p in gastric cancer. Mol. Cancer.

[14] Gagnon KT, Li L, Chu Y, Janowski BA, Corey DR (2014). RNAi factors are present and active in human cell nuclei. Cell Rep.

[15] Hanahan D, Weinberg RA (2011). Hallmarks of cancer: the next generation. Cell.

[16] Song IS (2009). Human ZNF312b promotes the progression of gastric cancer by transcriptional activation of the K-ras gene. Cancer Res..

[17] Liang C (2016). Energy sources identify metabolic phenotypes in pancreatic cancer. Acta Biochim. Biophys. Sin. (Shanghai)..

[18] Mercer TR, Mattick JS (2013). Structure and function of long noncoding RNAs in epigenetic regulation. Nat. Struct. Mol. Biol..

[19] Wilusz JE, Sunwoo H, Spector DL (2009). Long noncoding RNAs: functional surprises from the RNA world. Genes Dev..

[20] Batista PJ, Chang HY (2013). Long noncoding RNAs: cellular address codes in development and disease. Cell.

[21] Ota T (2004). Complete sequencing and characterization of 21,243 full-length human cDNAs. Nat. Genet..

[22] Glover AR (2015). Long noncoding RNA profiles of adrenocortical cancer can be used to predict recurrence. Endocr. Relat. Cancer.

[23] Chen N (2016). Long non-coding RNA FEZF1-AS1 facilitates cell proliferation and migration in colorectal carcinoma. Oncotarget.

[24] Shimizu T (2010). Zinc finger genes Fezf1 and Fezf2 control neuronal differentiation by repressing Hes5 expression in the forebrain. Development.

[25] Song IS (2011). Human ZNF312b oncogene is regulated by Sp1 binding to its promoter region through DNA demethylation and histone acetylation in gastric cancer. Int. J. Cancer.

[26] Prensner JR, Chinnaiyan AM (2011). The emergence of lncRNAs in cancer biology. Cancer Discov..

[27] Jiang H (2017). Long noncoding RNA CRNDE stabilized by hnRNPUL2 accelerates cell proliferation and migration in colorectal carcinoma via activating Ras/MAPK signaling pathways. Cell Death Dis..

[28] Vander HM, Cantley LC, Thompson CB (2009). Understanding the Warburg effect: the metabolic requirements of cell proliferation. Science.

[29] Liang C (2017). ARF6, induced by mutant Kras, promotes proliferation and Warburg effect in pancreatic cancer. Cancer Lett..

[30] Ohara Y (2013). Histological and prognostic importance of CD44(+) /CD24(+) /EpCAM(+) expression in clinical pancreatic cancer. Cancer Sci..

