# Supplementary material for: FEZF1-AS1/miR-107/ZNF312B axis facilitates progression and Warburg effect in pancreatic ductal adenocarcinoma
Source: Cell Death Dis. 2018 Jan 18;9(2):34. doi: 10.1038/s41419-017-0052-1 (PMC5833349; doi:10.1038/s41419-017-0052-1)
Supplement: Supplementary file 1 — Table S1 [file 41419_2017_52_MOESM1_ESM.docx]

**Table S1. Primers for qRT-PCR.**

| **Primer Name** | **Direction*** | **Sequence (5’ to 3’)** |
| --- | --- | --- |
| FEZF1-AS1 | F | TTAGGAGGCTTGTTCTGTGT |
|  | R | GCGCAGGTACTTAAGAAAGA |
| ZNF312B | F | CAGGCACAAGATCATTCACACGCAG |
|  | R | CCCTTTTTGATGAAACCCTTTGCCACAG |
| GAPDH | F | ACAGTCAGCCGCATCTTCT |
|  | R | GACAAGCTTCCCGTTCTCAG |
| miR-107 | F  R | GGAGCAGCATTGTACAGG  CAGTGCGTGTCGTGGA |
| U6 | F  R | GCTTCGGCAGCACATATACTAAAAT  CGCTTCACGAATTTGCGTGTCAT |

***F: forward; R: reverse.**
