# Supplementary material for: FEZF1-AS1/miR-107/ZNF312B axis facilitates progression and Warburg effect in pancreatic ductal adenocarcinoma
Source: Cell Death Dis. 2018 Jan 18;9(2):34. doi: 10.1038/s41419-017-0052-1 (PMC5833349; doi:10.1038/s41419-017-0052-1)
Supplement: Supplementary file 2 — Table S2 [file 41419_2017_52_MOESM2_ESM.docx]

**Table S2. Oligonucleotide sequences for this study.**

| **Name** | **Direction*** | **Sequence (5’ to 3’)** |
| --- | --- | --- |
| **miRNA**  miR-107 mimic |  | AGCAGCAUUGUACAGGGCUAUCA |
|  |  | AUAGCCCUGUACAAUGCUGCUUU |
| NC mimic |  | UUCUCCGAACGUGUCACGUTT |
|  |  | ACGUGACACGUUCGGAGAATT |
| miR-107 inhibitor |  | UGAUAGCCCUGUACAAUGCUGCU |
| NC inhibitor |  | CAGUACUUUUGUGUAGUACAA |
| **si-RNA** |  |  |
| si-FEZF1-AS1 #1 | F | GGCGAACAAUUAGAAGAUATT |
|  | R | UAUCUUCUAAUUGUUCGCCTT |
| si-FEZF1-AS1 #2 | F | GUCGCACUUUCAUCCACAATT |
|  | R | UUGUGGAUGAAAGUGCGACTT |
| **si-****FEZF1-AS1 #3** | F | CCCACGAAGUUUAAAGCAUTT |
| **most efficiency** | R | AUGCUUUAAACUUCGUGGGTT |
| si-FEZF1-AS1 #4 | F | GGUUACUGCAAUUCGAAAUTT |
|  | R | AUUUCGAAUUGCAGUAACCTT |
| si-ZNF312B #1 | F | GGGUUUCUGCAGGAACUUUGATT |
|  | R | UCAAAGUUCCUGCAGAAACCCTT |
| **si-ZNF312B #2** | F | GCACAAGAUCAUUCACACGCATT |
| **most efficiency** | R | UGCGUGUGAAUGAUCUUGUGCTT |
| si-NC | F | UUCUCCGAACGUGUCACGUTT |
|  | R | ACGUGACACGUUCGGAGAATT |
| **sh-RNA** |  |  |
| **sh-FEZF1-AS1 #1**  **most efficiency** | F | CCGGCCCACGAAGTTTAAAGCATAACTCGAGTTATGCTTTAAACTTCGTGGGTTTTTG |
|  | R | AATTCAAAAACCCACGAAGTTTAAAGCATAACTCGAGTTATGCTTTAAACTTCGTGGG |
| sh-FEZF1-AS1 #2 | F | CCGGGTCGCACTTTCATCCACAAAACTCGAGTTTTGTGGATGAAAGTGCGACTTTTTG |
|  | R | AATTCAAAAAGTCGCACTTTCATCCACAAAACTCGAGTTTTGTGGATGAAAGTGCGAC |
| sh-NC | F | CCGGTTCTCCGAACGTGTCACGTAACTCGAGTTACGTGACACGTTCGGAGAATTTTTG |
|  | R | AATTCAAAAATTCTCCGAACGTGTCACGTAACTCGAGTTACGTGACACGTTCGGAGAA |

***F: forward; R: reverse.**
