# Supplementary material for: FEZF1-AS1/miR-107/ZNF312B axis facilitates progression and Warburg effect in pancreatic ductal adenocarcinoma
Source: Cell Death Dis. 2018 Jan 18;9(2):34. doi: 10.1038/s41419-017-0052-1 (PMC5833349; doi:10.1038/s41419-017-0052-1)
Supplement: Supplementary file 3 — marked-up version of Supplementary Methods [file 41419_2017_52_MOESM3_ESM.docx]

**Supplementary Methods**

**Microarray analysis**

Transcriptomic analysis was carried out using a microarray (Arraystar Human LncRNA Microarrays V3; Agilent Technology, Santa Clara, USA), which targets 27958 Entrez protein-coding genes and 7419 lncRNAs. Total RNA was extracted and mRNA was purified using the mRNA-ONLY™ Eukaryotic mRNA Isolation Kit (Epicentre). Total RNA was fragmented, labeled (One-Color, Cy3, Agilent), purified and hybridized with probes in Hybridization Chamber gasket slides(Agilent). The slides were then washed and scanned with an Agilent Microarray Scanner. The raw data were extracted with Agilent Feature Extraction software (Agilent). This software used the robust multi-array average algorithm to adjust the background signals. Normalized data were obtained after performing the quantile method of intra-microarray normalization and the median method of baseline transformation between the microarrays. The microarray platform and data were submitted to the Gene Expression Omnibus public database at the National Center for Biotechnology Information (GEO, http://www.ncbi.nlm.nih.gov/geo/, ID: GSE61166).

**Cell culture**

Human pancreatic cancer cell lines (PANC-1, Capan-2, SW1990, MIAPaCa-2 and BxPC-3) were purchased from American Type Culture Collection (ATCC, Manassas, USA) and the immortal human pancreatic duct epithelial cell line (HPDE6-C7) was a kind gift from Prof. SN Zhang (Sun Yat-sen University, Guangdong, China). Cells were maintained in Dulbecco’s modified Eagle’s medium (DMEM; GIBCO-BRL; Invitrogen, CA, USA) or RPMI 1640 (GIBCO-BRL; Invitrogen, CA, USA) supplemented with 10% fetal bovine serum (FBS), 100 U/ml penicillin, and 100 mg/ml streptomycin and cultured at 37˚C in humidified air with 5% CO_2_.

**RNA extraction and quantitative real-time PCR (qRT-PCR)**

Total RNA was extracted from tissues or cultured cell lines using TRIzol reagent (Invitrogen, San Diego, CA, USA) following the manufacturer's instructions. RNA concentration was measured with NanoDrop ND-2000 spectrophotometers (Life Technologies, CA, USA) and then the total RNA (500 ng) was then transcribed to cDNA in a final volume of 10 ml using oligodT primers and SuperScript II reverse transcriptase (Invitrogen, San Diego, CA, USA). Real-time PCR was performed using SYBR Green reaction mix (Qiagen, Germany) and analyzed on a Roche Light-Cycler system (Roche, Basel, Switzerland). The results were normalized to the expression of glyceraldehyde-3-phosphate dehydrogenase (GAPDH). All the primer sequences were listed in Table S1. The qRT-PCR data were analyzed and expressed relative to CT (cycle threshold) values. The qRT-PCR results were analyzed and showed as the fold change (2^-∆∆CT^). For expressions in tissues, the levels were firstly normalized to GAPDH expression as △CT and then compared with one of the tissues and converted to the fold change (2^-∆∆CT^). To analyze the clinical significance of FEZF1-AS1, tissues from 94 PDAC patients were divided into two groups - the high and low expression group - depending on the fold change (2^-∆∆CT^) of FEZF1-AS1 by using the median expression value. For the analysis of the expression in cells, the levels were compared with the controls and converted to the fold change (2^-∆∆CT^). The quantitative PCR reaction for each sample was repeated in triplicate.

**Fluorescent in situ hybridization (FISH)**

A locked nucleic acid (LNA) probe with complementarity to a section of FEZF1-AS1 was labeled with 5-Carboxyfluorescein (FAM), which was synthesized by Biosense Bioscience Co.Ltd (Guangzhou, China). After dewaxing and rehydration, the samples were digested with proteinase K, denatured with formamide, hybridized with the FEZF1-AS1 probe at 42°C overnight. DAPI was used for counterstaining the nuclear subsequently. Samples were analyzed in a drop of fluorescence decay mounting medium under a fluorescence microscope. Excitation wavelengths used were at 360nm for DAPI, 490nm for FEZF1-AS1 with emissions detected at appropriate wavelengths. Negative control was a scramble LNA probe. The [probe sequence](http://www.baidu.com/link?url=DF8JkoxGmezjhwNOBn3-EBhpCx05DECPknt91HP3Zci8zblrzW5e-uM6x1AoKv3U8cREUD-tiuNisviBZ8UwPF5iT_opD0zGK4r3nGbVpai2nA-QLZs4LDqWoxTOgF68) of FEZF1-AS1 is as following: aatttctattatcgacttttatttcatgggatcacttaagaaagtttgaaaataacataaaaaatgctttaaacttcgtgggtagtgtcaaggaaacagtcgaaccgtgtcttgaatatggttgttttgtgtgtgtgtctgcttgtttctaattttggccacaaacacaaaatgtctttaaaattgtccagggactgctgcagaacgctaatcaatcaattgcacgaaaacaggttttttctattattggaggcaagtatttcagtgtagagcaatagagcatgtttgggctatcttctaattgttcgccttccagatttcagtaacacccttgtaagtgacaagcaaaactttgctgggttttgttgcttgacgctgttgctccacagtaaaggttggagaaactgggttacttgttttataaacttctttttttccataaagtccaaccctgagtcatagcctctcagtctggagggacacacctcacaggc.

**Cell transfection and viral infection**

For transient knockdown experiments, the following small interfering RNAs (siRNAs) were as follows: FEZF1-AS1 siRNA (si- FEZF1-AS1), ZNF312B siRNA (si-ZNF312B), and synthetic sequence-scrambled siRNA (si-NC) were purchased from GenePharma Co. (Shanghai, China). The siRNAs were transfected into cells using Lipofectamine 3000 (Life Technologies, CA, USA) according to the manufacturer’s instruction. After 48 hours, the efficiency of siRNA knockdown was confirmed via qRT-PCR. For stable knockdown of FEZF1-AS1, FEZF1-AS1 shRNA (sh-FEZF1-AS1) and scrambled control RNA (sh-NC) were inserted into the lentiviral vector (pLKO.1-puro vector). 72 hours after transfection of 293T cells, the viral supernatants were collected. Using a LentiX™ Concentrator overnight at 4°C (Clontech, Mountain View, CA, USA), lentiviral particles were concentrated and titered to 10^9^ TU/ml (transfection unit/ml). PANC-1 cells (5×10^5^ cells/well) were seeded in six-well culture plates and maintained in DMEM with 10% FBS and then infected with virus and polybrene 24 hours later. Positive clones were screened with puromycin (2μg/ml and 5μg/ml, respectively) for 2-3 weeks to establish the following new stable cell lines: PANC-1-sh-FEZF1-AS1 (FEZF1-AS1 stable knockdown) and PANC-1-sh-NC (negative control). All oligonucleotide sequences were listed in Table S2.

**Cell proliferation assay and colony formation assay**

Cell proliferation was measured using a cell counting kit-8 (CCK-8) assay according to the manufacturer’s instructions. Cells were seeded in 96-wells plates (2x10^3^ cells/well), at the appropriate time (0, 24, 48, 72 and 96 hours), CCK-8 solution (10 μL, Dojindo Molecular Technologies, Kyushu, Japan) was added and the cells were incubated for 4 hours at 37^o^C. Absorbance was measured at a wavelength of 450 nm. The assays were repeated three times.

For colony formation assay, a total of 600 cells transfected with si-FEZF1-AS1, si-ZNF312B or si-NC combined with miR-107 inhibitor or mimic were seeded in 6-well plates and maintained in media containing 10% FBS at 37^o^C, which was replaced every 3 days. After 14 days, colonies were fixed with methanol and stained with 0.1% crystal violet ([Sigma-Aldrich](http://www.sigmaaldrich.com/catalog/product/sigma/ht90132?lang=en&region=US), Milwaukee, USA). Visible colonies were then manually counted. Wells were measured in triplicate for each treatment group.

**Cell apoptosis and cell cycle analysis**

For cell apoptosis, PANC-1 and Capan-2 cells (5×10^4^ cells) were collected via trypsin digestion, washed with cold phosphate-buffered saline (PBS), and resuspended in Annexin V binding buffer. AV-FITC（Becton Dickinson Biosciences, USA）at a final concentration of 1 µg/ml and 250 ng of PI were added to a mixture containing 100µL each of cell resuspension and binding buffer (Becton Dickinson Biosciences, USA). The mixture was incubated in the dark for 15 minutes at room temperature. Cells were washed once with binding buffer and resuspended in 400 µL of binding buffer prior to flow cytometric analysis using a BD FAC Scan Flow Cytometer (BD, Mountain View, USA).

For cell-cycle analysis, PANC-1 and Capan-2 cells (5×10^4^ cells) were collected and washed three times with PBS. Cells were then incubated in propidium iodide (PI) staining solution (RNase A 100 ug/mL and PI 500 ug/mL) for 30 minutes at 4°C, and cells were analyzed by flow cytometry. Each study was repeated at least three times.

**Wound healing assay and transwell assay**

Cells were incubated with normal cell growth medium in 6-well plates. Once cultures reached 85% confluency, the cell layer was scratched with a 10 μl sterile pipette tip and washed with culture medium, then exchanged with medium containing 1% FBS cultured for 48 hours. To prevent cell proliferation, which could confound the analysis of cell migration into the wound, cells were preincubated with mitomycin C (10 μg/ml) for 1 hour at 37 °C. At different two points (0 hour, 32 hours), images of the plates were acquired using a microscope. Experiments were performed at least three times.

Cell migration and invasion assays were performed using transwell chambers (24-well insert, 8 μm, Corning Costar Corp). Cells were harvested at 48 hours after transfection and then collected. 5 × 10^4^ cells were suspended in serum-free medium and placed in the uncoated (migration assay) or 1:8 diluted Matrigel-coated (invasion assay, BD Biosciences, NJ, USA) upper chamber. The lower chamber was filled with 500ul of medium containing 20% FBS. After incubation for 36 hours at 5% CO_2_ at 37°C, the cells remaining on the upper chamber were removed with cotton wool, cells migrating to the bottom surface of the membrane from the upper chamber were fixed with methanol, stained with 0.1% crystal violet solution, then were imaged and five random fields were counted. Experiments were performed at least three times.

**Western blot analysis**

Cells were washed in PBS and lysed using the protein extraction reagent RIPA (Invitrogen, Carlsbad, CA) supplemented with a protease inhibitor cocktail (Roche, Pleasanton, CA, USA) and PMSF (Roche). The protein concentration was calculated using a bicinchoninic acid protein assay kit (Pierce, Rockford, IL, USA). Equivalent amounts of proteins (35 μg) from each sample were electrophoresed on a 8% SDS–polyacrylamide gel (SDS–PAGE) and then transferred to a polyvinylidene fluoride membrane, blocked in 5% fat-free milk for 2 hours at room temperature, and incubated with the following specific primary antibodies: rabbit anti-human ZNF312B antibody (1:1000, #ab81251, Abcam) and rabbit anti-human GAPDH antibody (1:1000, #ab18162, Abcam). GAPDH was used as a loading control. They were then incubated with the following HRP-linked secondary antibody: goat anti-rabbit IgG (1: 5000; Cell Signaling Technology, Boston, USA). An ECL chemiluminescence kit (Pierce) was used to detect specific bands and autoradiograms were quantified by densitometry (Quantity One software, Bio-Rad, Hercules, CA, USA) using GAPDH as a control.

**Immunohistochemistry staining and scoring**

Paraffin-embedded samples of primary carcinomas were stained for ZNF312B. Sections were deparaffinized in xylene and rehydrated in a graded series of ethanol, followed by heat-induced epitope retrieval in citrate buffer (pH = 6.0). Antigen retrieval was performed in 10 mmol/L citrate buffer (pH = 6.0) in a microwave oven for 15 minutes. The activity of endogenous peroxidases was blocked by the addition of 3% hydrogen peroxide for 10 minutes at room temperature. Rabbit anti-human ZNF312B antibody (1:200, #ab81251, Abcam) was applied overnight at 4°C, and after washing three times in PBS, sections were immunostained with a goat anti-rabbit IgG (1: 5000; Cell Signaling Technology, Boston, USA) for 1 hour at 37°C. The slides were incubated with streptavidin-HRP conjugate complex for 45 minutes at 37°C. After rinsing three times in PBS, the sections were developed with 3, 3’-diaminobenzidine. Sections were counterstained with hematoxylin. Sections of skin tissues known to stain positive for ZNF312B were used as positive controls, and normal goat serum and PBS substituting the primary antibody were used as negative controls.

For evaluation and grading of ZNF312B staining results, a scoring criterion previously described by Ohara Y et al. was used [13]. Briefly, the staining intensity of ZNF312B was graded on a scale of 0–3 (0, none; 1, weak; 2, intermediate; and 3, strong). ZNF312B expression was assessed according to the percentage of staining as follows: 0 points for no staining; 1 point for <25% staining; 2 points for 26–50% staining; 3 points for 51–75% staining; and 4 points for 76–100% staining. The total score was calculated as the product of the scores for the intensity and positive rate of staining. Staining was assessed by two pathologists according to the scoring criteria. Cases with discrepancies were jointly reevaluated until a consensus was reached.

**Tumor formation assay in a nude mouse model**

The athymic BALB/c nude mice (4-6 weeks old) were purchased and maintained at the Laboratory Animal Center of Sun Yat-sen University in a specific pathenogen-free environment. Mice were given continuous access of food and water. The animal care and experimental protocols were approved by the institutional guidelines of Guangdong Province and by the Use Committee for Animal Care. PANC-1 cells stably transfected with sh-FEZF1-AS1 or sh-NC were cultured in six well plates for 48 hours. Then, the cells were collected, washed with phosphate-buffered saline and resuspended at 1 × 10^8^ cells/ml. A total of 100 μl of suspended cells with stable knockdown of FEZF1-AS1, or mock cells, were respectively injected subcutaneously into left and right bilateral hind leg of mice. At day 3 after the injection of tumor cells, the tumor growth was evaluated once every 3 days by measuring the length and the width with electronic calipers. The tumor volume was calculated using the following formula: V = (L × W^2^)/2 (V, volume; L, length diameter; W, width diameter). The mice were sacrificed by cervical dislocation at 27 days post injection, and tumors were collected for further study (weight measurement and RNA extraction). FEZF1-AS1 levels were determined by qRT-PCR.
