# Supplementary material for: FEZF1-AS1/miR-107/ZNF312B axis facilitates progression and Warburg effect in pancreatic ductal adenocarcinoma
Source: Cell Death Dis. 2018 Jan 18;9(2):34. doi: 10.1038/s41419-017-0052-1 (PMC5833349; doi:10.1038/s41419-017-0052-1)
Supplement: Supplementary file 4 — Supplementary Figures [file 41419_2017_52_MOESM4_ESM.docx]

**Figure S1** Differentially expressed lncRNAs in PDAC and chronic pancreatitis tissues. (A) The heat map of the top 50 differentially expressed lncRNA probes in PDAC and chronic pancreatitis tissues. (B) LncRNA FEZF1-AS1 was one of the most up-regulated lncRNAs in PDAC tissues compared with non-tumorous tissues.

**Figure S2** FEZF1-AS1 and ZNF312B over-expression is associated with clinicopathological characteristics and poor prognosis of PDAC patients. (A) Kaplan-Meier curves of overall survival in all patients with PDAC according to FEZF1-AS1 expression. The log-rank test was used to compare differences between groups. (B) Kaplan-Meier analysis of overall survival stratified by relative intensity of ZNF312B expression (Score 0, *n*=15; Score 1, *n*=22; Score 2, *n*=31; Score 3, *n*=26, *P*<0.001). The log-rank test was used to compare differences between groups. (C) Representative images of ZNF312B staining in PDAC tissues (score 0-3). ******* *P*<0.001.

**Figure S3** Prognostic nomogram with calibration and validation analysis. (A) Nomogram for predicting overall survival according to the prognostic factors from the results of multivariate analysis, which led to regrouping based on AJCC stage, neural invasion, ZNF312B expression and FEZF1-AS1 expression. (B-C) Calibration curve for predicting 3-year OS and 5-year OS patients outcomes. A plot along the 45-degree line would indicate a perfect calibration model in which the predicted probabilities were identical to the actual outcomes. OS, overall survival.

**Figure S4** Effects of FEZF1-AS1 or ZNF312B over-expression on the promotion of PDAC cell proliferation and colony formation could be diminished by miR-107 mimic *in vitro*.（A-B）The expression of FEZF1-AS1 or ZNF312B was over-expressed by transfecting of pcDNA3.1-FEZF1-AS1 or pcDNA3.1-ZNF312B into PANC-1 and Capan-2 cells. (C-D) The cell viability of pcDNA3.1-NC, pcDNA3.1-FEZF1-AS1, pcDNA3.1-NC+miR-107 mimic, pcDNA3.1-FEZF1-AS1+miR-107 mimic or pcDNA3.1-ZNF312B transfected PANC-1 and Capan-2 cells by CCK-8 assay. (E) The proliferation of pcDNA3.1-NC, pcDNA3.1-FEZF1-AS1, pcDNA3.1-NC+miR-107 mimic, pcDNA3.1-FEZF1-AS1+miR-107 mimic or pcDNA3.1-ZNF312B transfected PANC-1 and Capan-2 cells by colony formation assay. Values represented the mean ± SD from three independent experiments. ***** *P*<0.05, ****** *P*<0.01, ******* *P*<0.001, Student’s *t*-test.

**Figure S5** Effects of FEZF1-AS1 or ZNF312B knockdown on the promotion of PDAC cell apoptosis and G1 phase arrest could be rescued by miR-107 inhibitor *in vitro*. (A-B) The cell apoptotic rate of si-NC, si-FEZF1-AS1, si-NC+miR-107 inhibitior, si-FEZF1-AS1+miR-107 inhibitor or si-ZNF312B transfected PANC-1 and Capan-2 cells by flow cytometry analysis. (C-D) The cell cycle distribution of si-NC, si-FEZF1-AS1, si-NC+miR-107 inhibitor, si-FEZF1-AS1+miR-107 inhibitor or si-ZNF312B transfected PANC-1 and Capan-2 cells by flow cytometry analysis. Values represented the mean ± SD from three independent experiments. ***** *P*<0.05, ****** *P*<0.01, ******* *P*<0.001, Student’s *t*-test.

**Figure S6** Effects of FEZF1-AS1 or ZNF312B FEZF1-AS1 or ZNF312B over-expression on the inhibition of PDAC cell apoptosis and G1 phase arrest could be diminished by miR-107 mimic *in vitro*. (A-B) The cell apoptotic rate of pcDNA3.1-NC, pcDNA3.1-FEZF1-AS1, pcDNA3.1-NC+miR-107 mimic, pcDNA3.1-FEZF1-AS1+miR-107 mimic or pcDNA3.1-ZNF312B transfected PANC-1 and Capan-2 cells by flow cytometry analysis. (C-D) The cell cycle distribution of pcDNA3.1-NC, pcDNA3.1-FEZF1-AS1, pcDNA3.1-NC+miR-107 mimic, pcDNA3.1-FEZF1-AS1+miR-107 mimic or pcDNA3.1-ZNF312B transfected PANC-1 and Capan-2 cells by flow cytometry analysis. Values represented the mean ± SD from three independent experiments. ***** *P*<0.05, ****** *P*<0.01, ******* *P*<0.001, Student’s *t*-test.

**Figure S7** Effects of FEZF1-AS1 or ZNF312B over-expression on the promotion of PDAC cell migration and invasion could be diminished by miR-107 mimic *in vitro*. PDAC Cell line PANC-1 and Capan-2 were treated as in described in Materials and Methods. (A-B) The motility of PANC-1 and Capan-2 cells transfected with pcDNA3.1-FEZF1-AS1 or pcDNA3.1-ZNF312B compared with the controls by wound healing assay. (C-D) The migration and invasion of PANC-1 and Capan-2 cells transfected with pcDNA3.1-NC, pcDNA3.1-FEZF1-AS1, pcDNA3.1-NC+miR-107 mimic, pcDNA3.1-FEZF1-AS1+miR-107 mimic or pcDNA3.1-ZNF312B compared with the controls by transwell assay. Values represented the mean ± SD from three independent experiments. ***** *P*<0.05, ****** *P*<0.01, ******* *P*<0.001, Student’s *t*-test.

**Figure S8** Effects of FEZF1-AS1 or ZNF312B over-expression on the promotion of PDAC cell glycolytic capacity could be diminished by miR-107 mimic *in vitro*. (A-B) Ectopic FEZF1-AS1 expression facilitated the glycolytic capacity of PANC-1 and Capan-2 cells, while the miR-107 mimic could abrogate the promotion effect of FEZF1-AS1 over-expression on the glycolytic process in both PDAC cell lines, as reflected by ECAR analysis. (C-D) Ectopic ZNF312B expression promoted glycolytic capacity of PANC-1 and Capan-2 cells, as reflected by ECAR analysis. (E-F) Ectopic FEZF1-AS1 or ZNF312B expression promoted the glucose uptake and lactate production of PANC-1 and Capan-2 cells, while the miR-107 mimic diminished the effect of FEZF1-AS1 or ZNF312B over-expression on glucose uptake and lactate production in both PDAC cell lines. Intracellular glucose levels were measured and normalized based on protein concentration. (G) PANC-1 and Capan-2 cells expressing pcDNA3.1-FEZF1-AS1 or pcDNA3.1-ZNF312B combined with miR-107 mimic were cultured under normoxic conditions for 24 hours. Acidification of the culture medium was evaluated by visually inspecting the colour of the medium. ***** *P*<0.05, ****** *P*<0.01.
