# Supplementary figures and images for: FEZF1-AS1/miR-107/ZNF312B axis facilitates progression and Warburg effect in pancreatic ductal adenocarcinoma
Source: Cell Death Dis. 2018 Jan 18;9(2):34. doi: 10.1038/s41419-017-0052-1 (PMC5833349; doi:10.1038/s41419-017-0052-1)

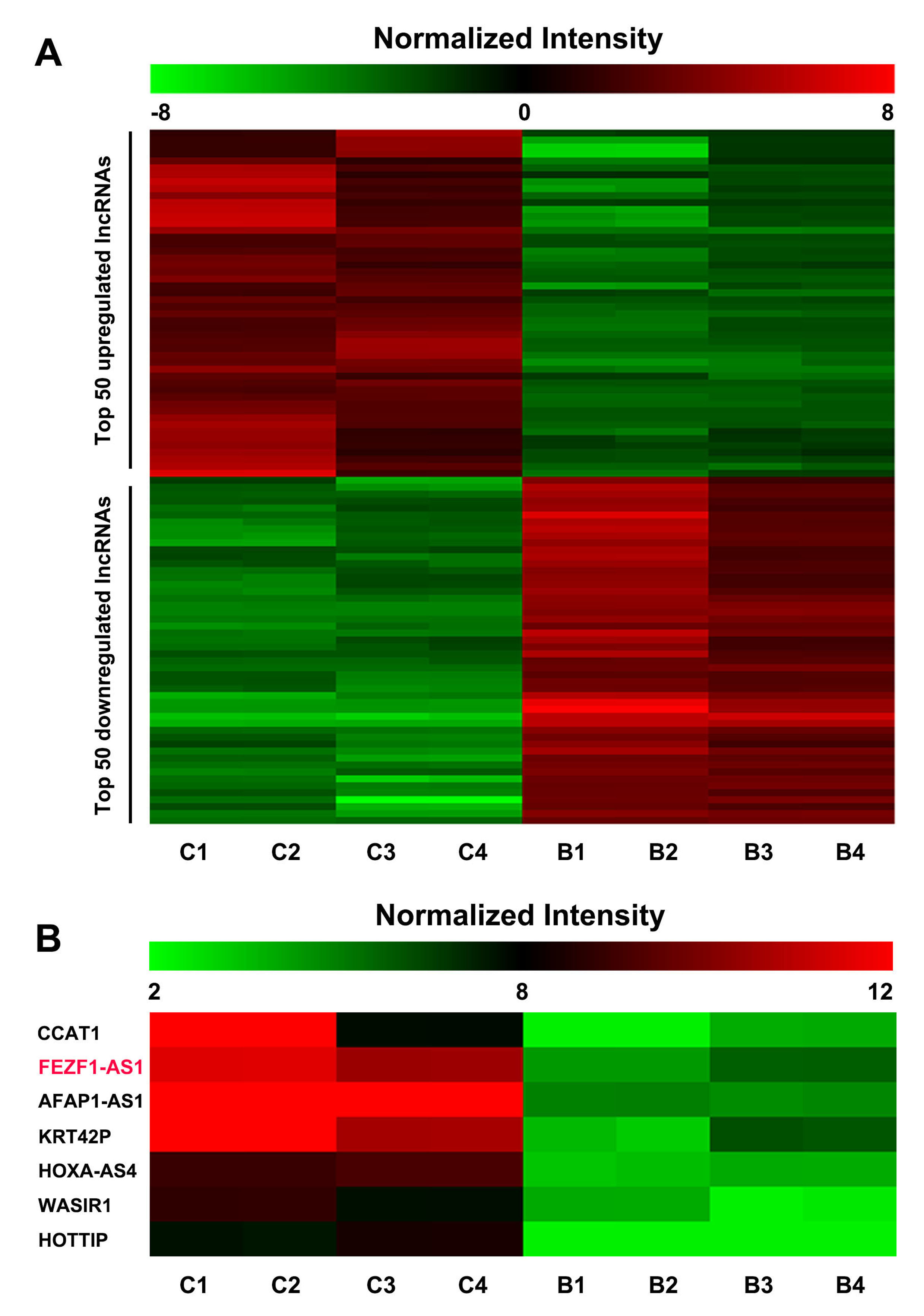

Supplement: Supplementary file 5 — Figure S1 [file 41419_2017_52_MOESM5_ESM.jpg]

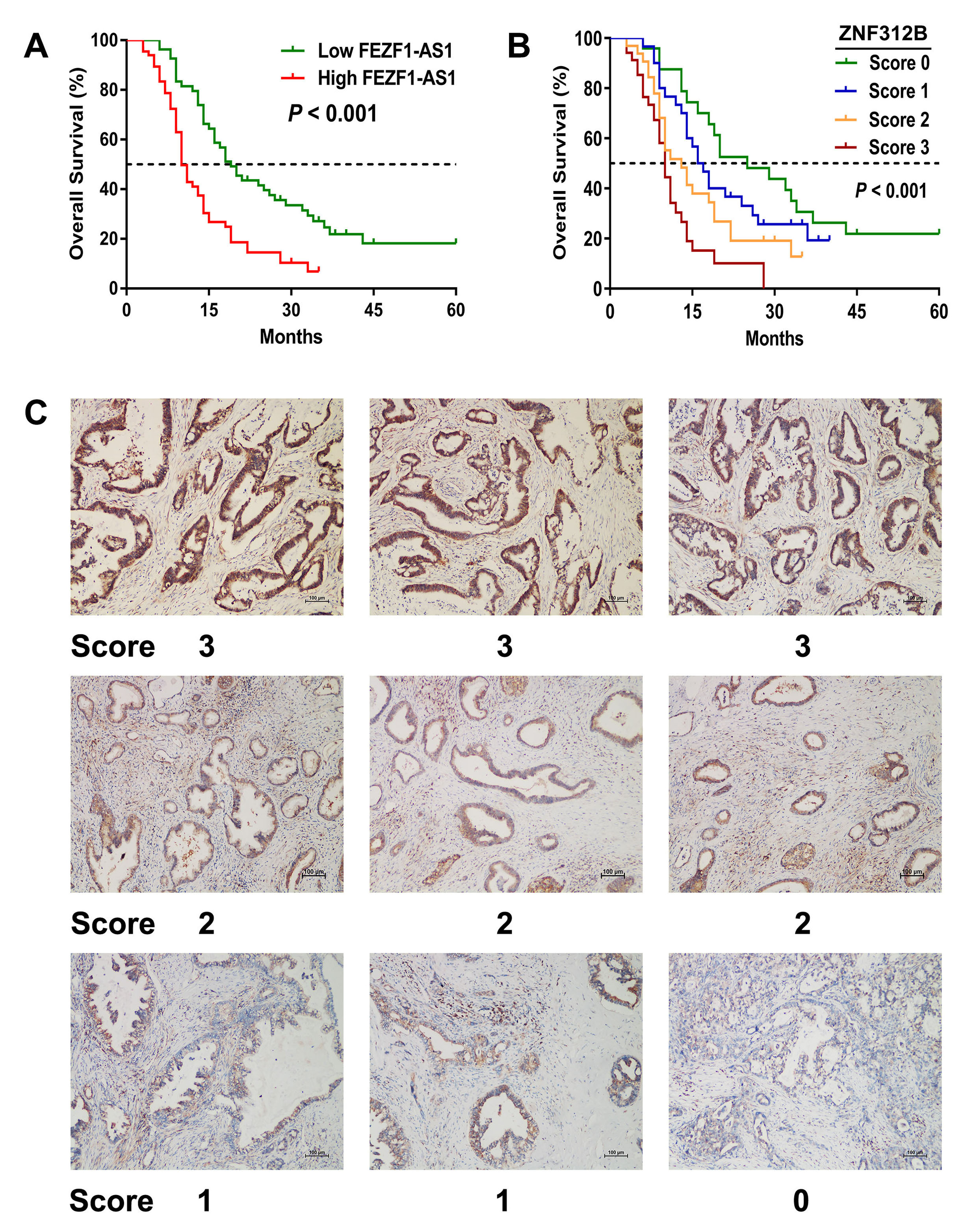

Supplement: Supplementary file 6 — Figure S2 [file 41419_2017_52_MOESM6_ESM.jpg]

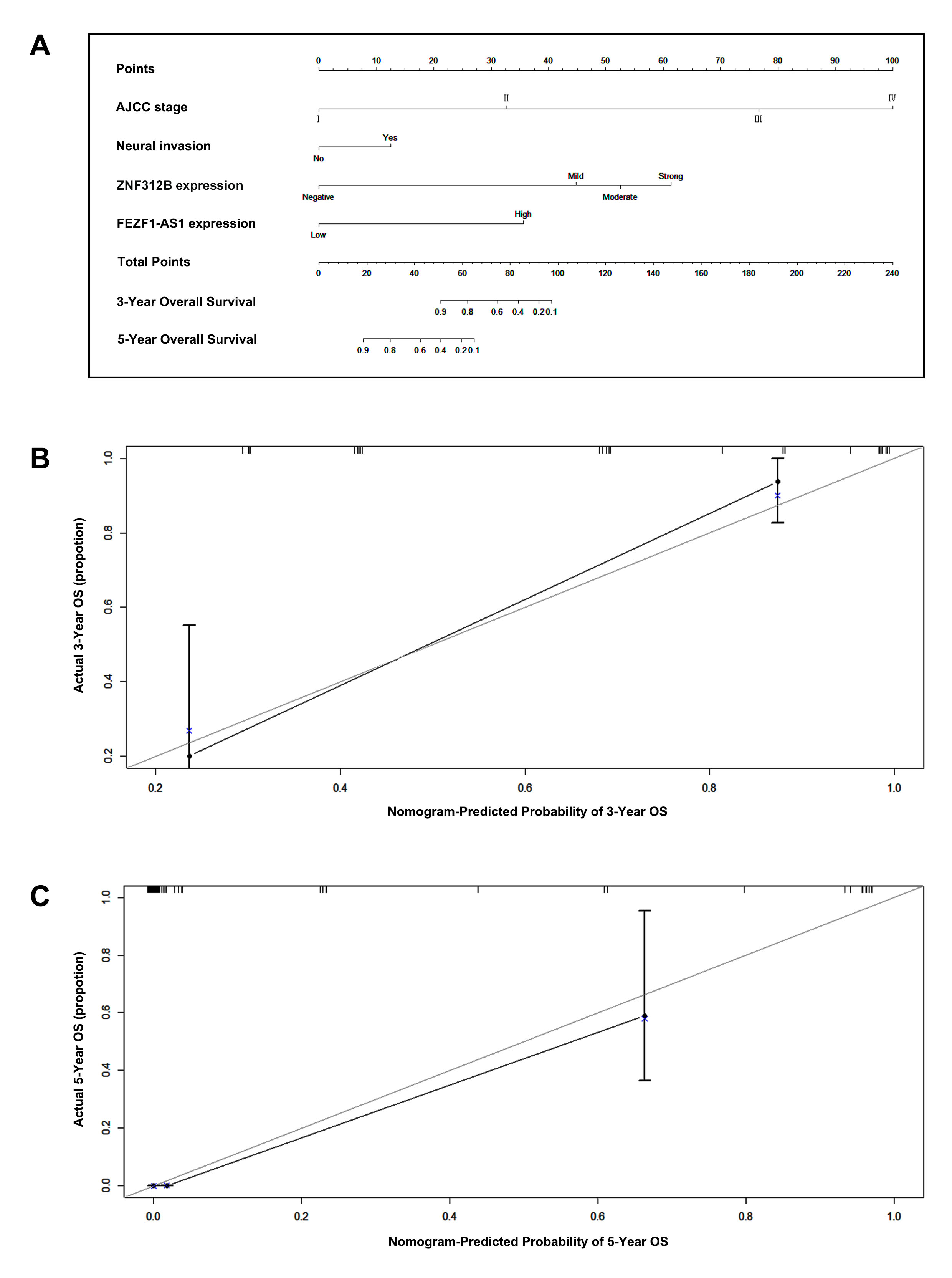

Supplement: Supplementary file 7 — Figure S3 [file 41419_2017_52_MOESM7_ESM.jpg]

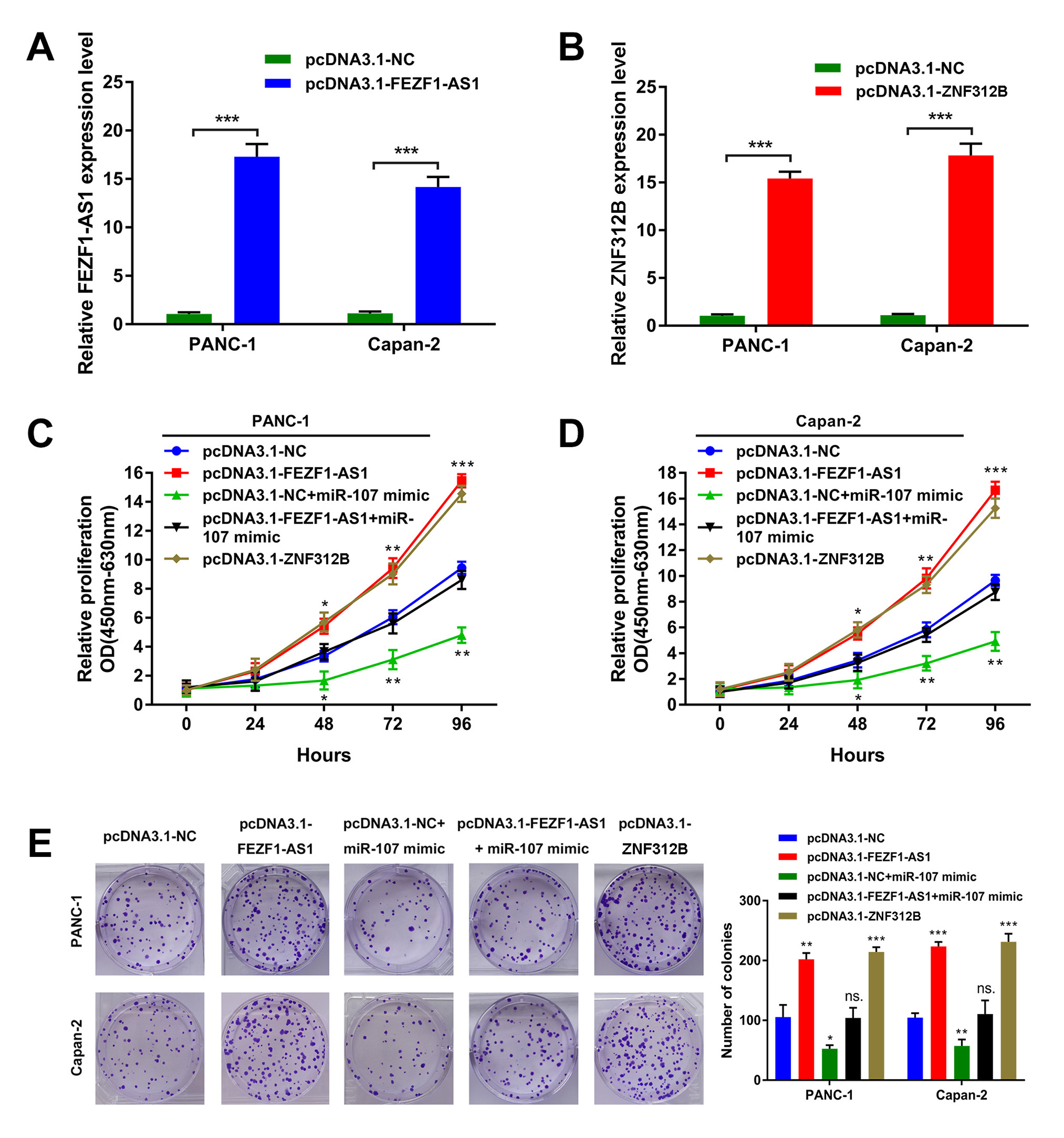

Supplement: Supplementary file 8 — Figure S4 [file 41419_2017_52_MOESM8_ESM.jpg]

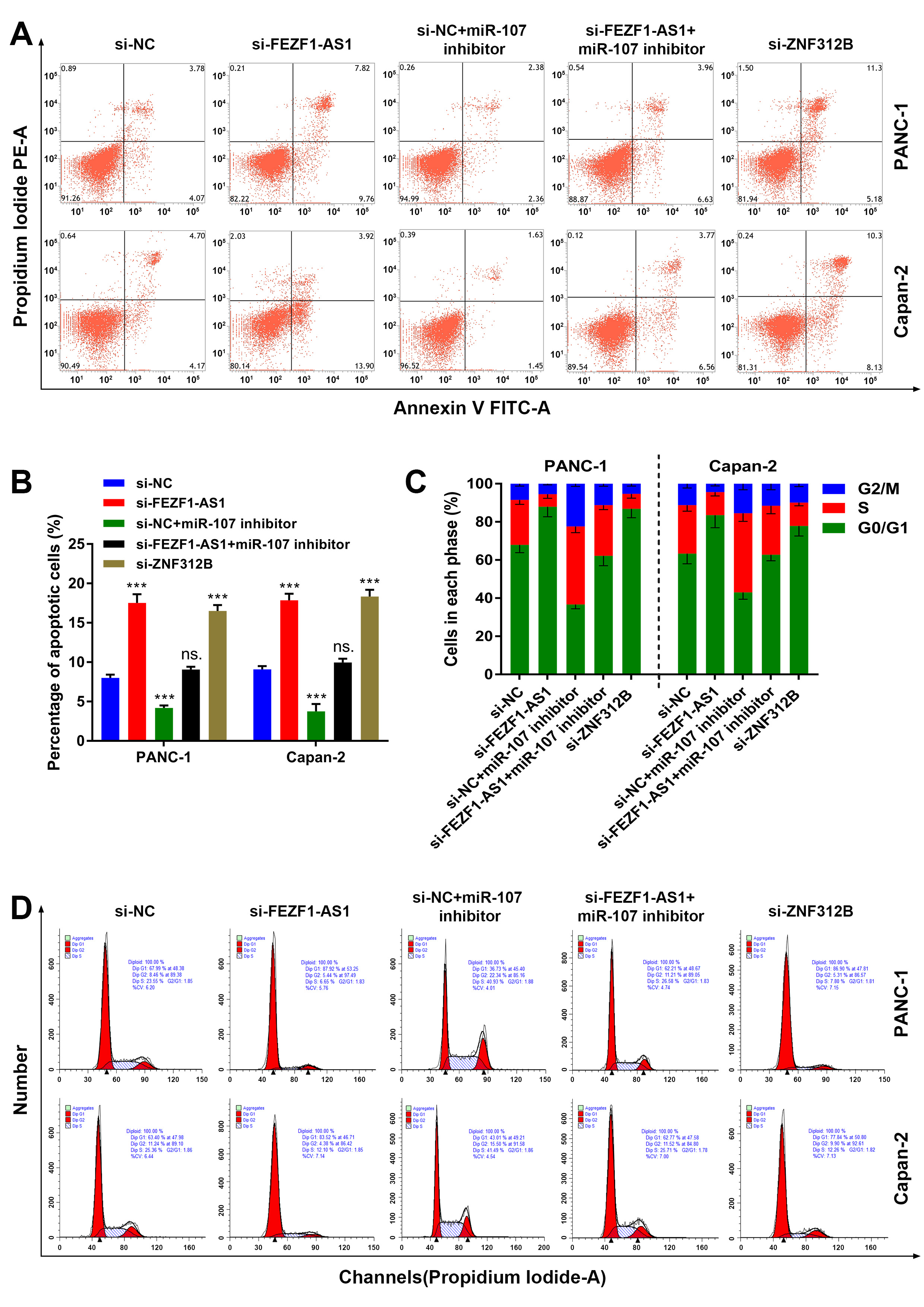

Supplement: Supplementary file 9 — Figure S5 [file 41419_2017_52_MOESM9_ESM.jpg]

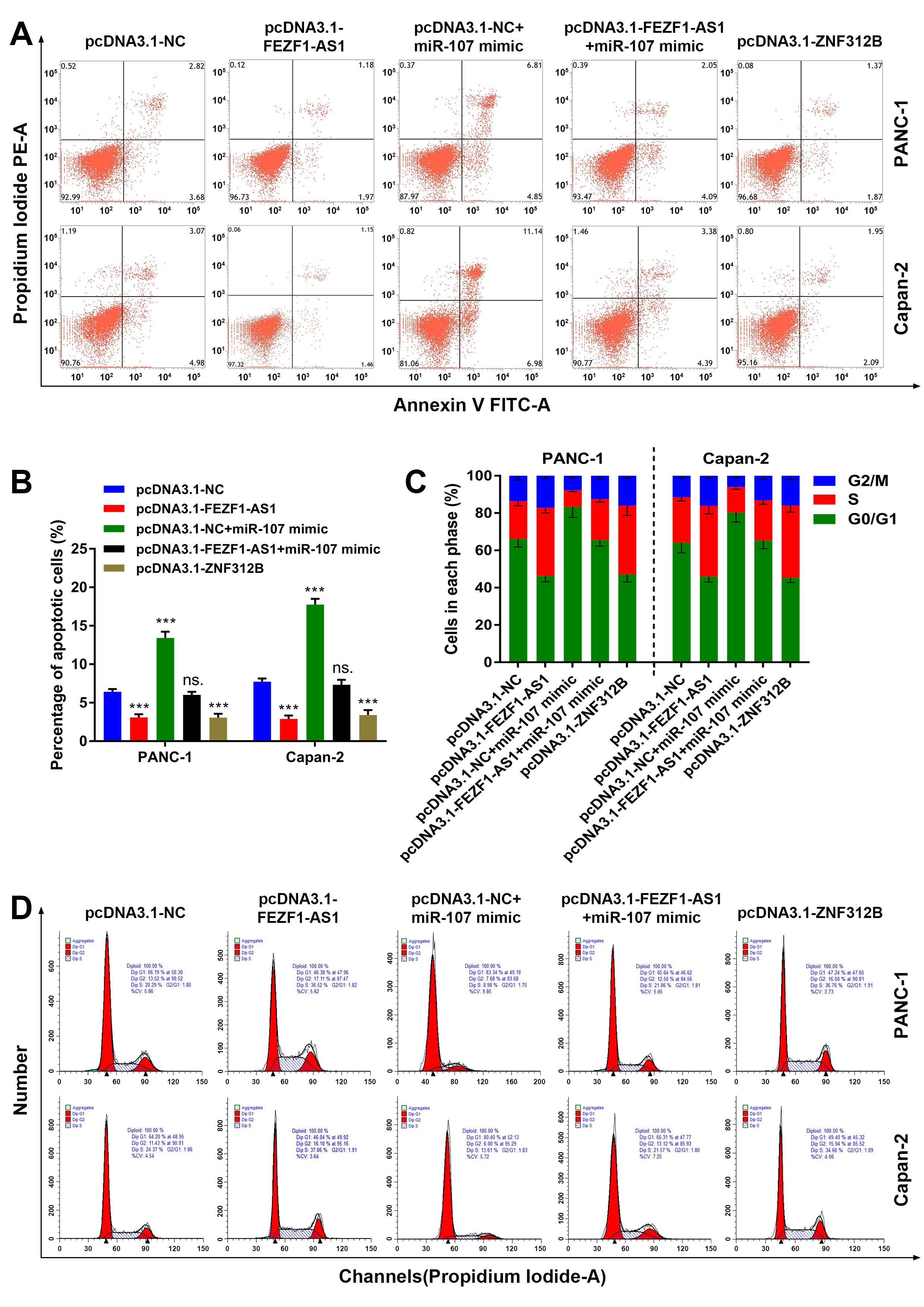

Supplement: Supplementary file 10 — Figure S6 [file 41419_2017_52_MOESM10_ESM.jpg]

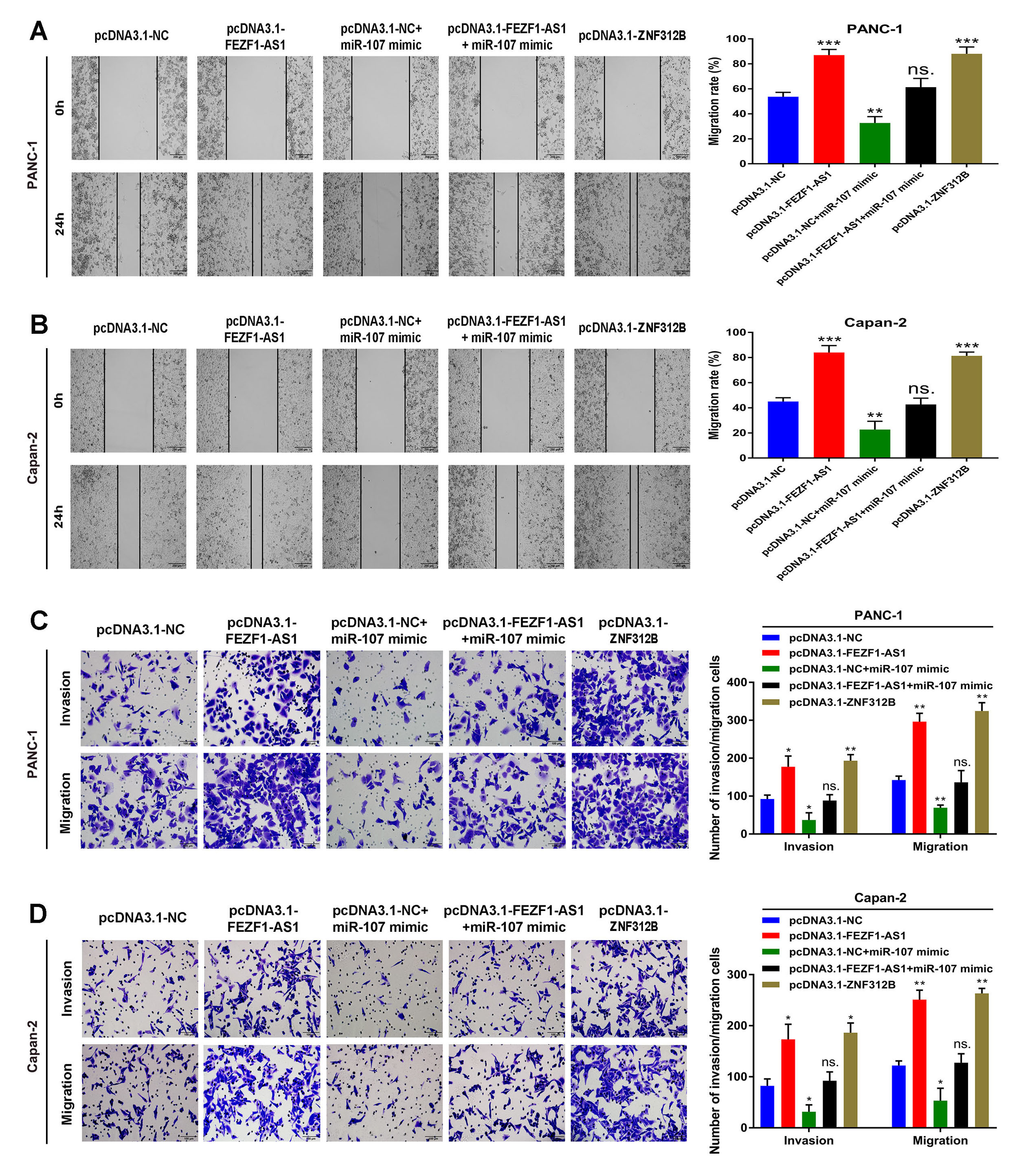

Supplement: Supplementary file 11 — Figure S7 [file 41419_2017_52_MOESM11_ESM.jpg]

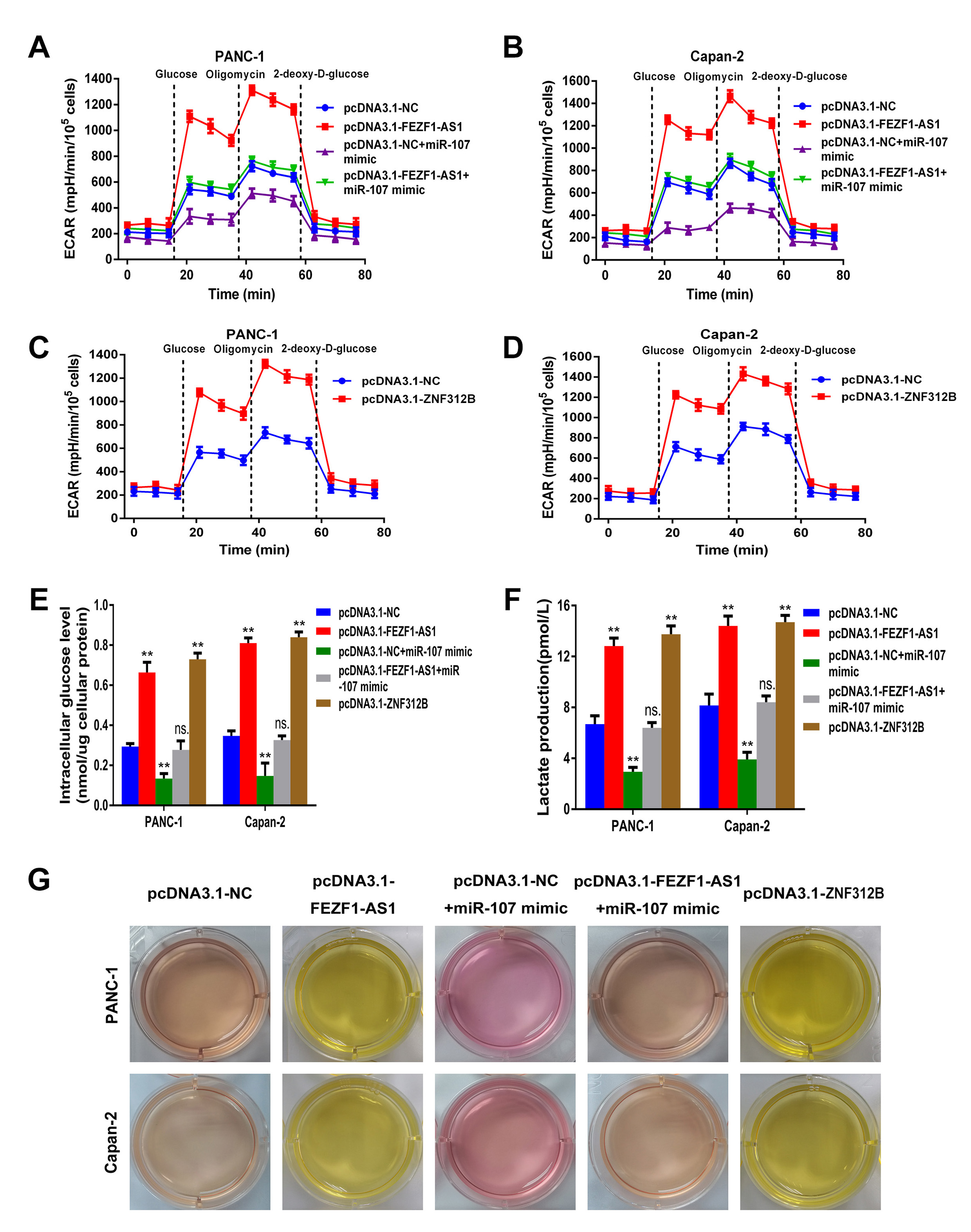

Supplement: Supplementary file 12 — Figure S8 [file 41419_2017_52_MOESM12_ESM.jpg]
